# Transition experiences of patients and families upon discharge from intensive care: a scoping review*

**DOI:** 10.1590/1518-8345.7919.4762

**Published:** 2025-12-01

**Authors:** Paula Buchs Zucatti, Clediane Rita Portalupi da Trindade, Rosana Evangelista-Poderoso, Edinêis de Brito Guirardello, Maria Alice Dias da Silva Lima

**Affiliations:** 1 Universidade Federal do Rio Grande do Sul, Escola de Enfermagem e de Saúde Coletiva, Porto Alegre, RS, Brazil. Universidade Federal do Rio Grande do Sul Escola de Enfermagem e de Saúde Coletiva RS Porto Alegre Brazil; 2 Hospital de Clínicas de Porto Alegre, Centro de Tratamento Intensivo, Porto Alegre, RS, Brazil. Hospital de Clínicas de Porto Alegre Centro de Tratamento Intensivo RS Porto Alegre Brazil; 3 Universidade Estadual de Campinas, Biblioteca da Faculdade de Ciências Médicas, Campinas, SP, Brazil. Universidade Estadual de Campinas Biblioteca da Faculdade de Ciências Médicas SP Campinas Brazil; 4 Universidade Estadual de Campinas, Faculdade de Enfermagem, Campinas, SP, Brazil. Universidade Estadual de Campinas Faculdade de Enfermagem SP Campinas Brazil; 5 Scholarship holder at the Conselho Nacional de Desenvolvimento Científico e Tecnológico (CNPq), Brazil. Scholarship holder at the Conselho Nacional de Desenvolvimento Científico e Tecnológico Brazil

**Keywords:** Patient Discharge, Patient-Centered Care, Continuity of Patient Care, Transitional Care, Patient Participation, Patient Transfer

## Abstract

to map available evidence on the experiences of adult patients and family members regarding the transition of care between intensive care and inpatient units.

scoping review conducted according to Joanna Briggs Institute guidelines. Primary studies, reviews, dissertations, and theses published in Portuguese, English, or Spanish were included, with no time limit. The final search was conducted on health portals and databases and in digital libraries. The selection was performed by two reviewers in a blind and independent manner. The data were extracted using a script and presented in figures, descriptive and deductive qualitative analysis, categorizing the experiences between the stages of assessment, planning, execution, and follow-up after discharge.

thirty articles comprised the sample. The experiences mapped highlight the transition as a critical moment in hospitalization, marked by emotional impact and informational weaknesses. Reports of unpreparedness, feelings of abandonment, and communication failures compromise the continuity of care and the adaptation of patients and family members.

the experiences described point to the transition of care between intensive care and inpatient units as a complex and multifaceted process. The gaps identified require structured communication strategies, timely information, and alignment between units.

## Highlights

**(1)** The transition between intensive care and inpatient care is complex and challenging. **(2)** Patients and family members experience an abrupt reduction and discontinuity of care. **(3)** Inaccurate or insufficient communication is present at all stages of the process. **(4)** The presence and involvement of family members as a source of emotional and informational support.

## Introduction

The transfer from the Intensive Care Unit (ICU) to the inpatient unit represents a significant change, marking an important transition process for the patient and their family. While the change is a specific and time-limited event, the transition is a continuous process, characterized by a series of responses. During this period, individuals face unfamiliar contexts, sensations, and emotions, in addition to dealing with uncertainties about the future, which places them in a vulnerable position^(1)^.

The way a person interprets change, its meaning, and the availability of support and resources directly impact their experiences, which can either help or hinder their recovery^(1)^. The patient experience is the sum of all interactions shaped by organizational culture, which influence their perceptions throughout the continuum of care^(2)^.

Preparing patients and their families to deal with the transition between different levels of care, as well as ensuring continuity of care until the changes are incorporated into their daily lives, plays a key role. This experience encompasses their understanding of the discharge process, their level of knowledge and engagement, the actions of professionals that condition their experiences, and whether or not their expectations are met^(1)^.

In addition to the individual’s situational fragility, the transfer from intensive care to regular care is recognized as a critical point during hospitalization. This process is especially susceptible to failures, whether in the transfer of the patient or in the transfer of information between sectors of the healthcare system. In this sense, the proper transition of care, understood as a structured set of actions performed by health professionals to ensure the coordination and continuity of care, is essential^(3-5)^.

In the high-acuity ICU setting, fragmentation of care has been associated with negative outcomes, such as increased stress and dissatisfaction among patients and families, longer hospital stays, adverse events, higher hospital costs, readmissions, and deaths^(6-10)^. Furthermore, individuals’ experiences in the transition between intensive care and inpatient units are influenced by the planning, communication, and execution of this process^(11)^.

A recent survey of intensive care professionals and managers revealed gaps between the moment when patients no longer require intensive care and the moment when they can safely transition to regular care^(12)^. This discrepancy reinforces the perception, reported by nurses in inpatient units, that there are “two distinct worlds”: while intensive care professionals have high expectations of the management capabilities of the inpatient team, these demands can pose a threat to patient safety^(13-14)^. In addition, patients themselves describe difficulties throughout their recovery, highlighting the lack of clear and accessible information, as well as the absence of spaces for listening and clarifying doubts, which compromises the care experience during this critical period^(15-17)^.

A structured approach involves sequential steps for the transition of care between intensive care and inpatient units. These steps include: 1) assessment for discharge using specific scores to determine severity, risks, and clinical evolution; 2) discharge planning, which consists of summarizing health problems, reviewing care goals, medication reconciliation, and providing detailed guidance to the patient and family about the care received, the care planned, the discharge process, and the destination; 3) execution of discharge, which involves deciding on the appropriate time for transfer, identifying the receiving team, and effective communication between the parties involved; and 4) follow-up after discharge, with a member of the ICU team providing care to the patient in the inpatient unit, ensuring continuity of care and facilitating adaptation to the new environment^(8)^.

Although the experiences of patients and their families in the context of ICU discharge have been widely discussed in the literature over the past few years^(6,8)^, Addressing them based on the stages of transition from intensive care to inpatient care is an innovative approach. This approach allows us to identify critical points in the process and determine the most appropriate time for specific interventions, contributing to improving the quality of transitions and mitigating the negative impacts of this experience in the immediate period after discharge.

Based on the above, a preliminary search was conducted in the Joanna Briggs Institute (JBI) Evidence Synthesis, PubMed^®^, and the Cumulative Index to Nursing and Allied Health Literature (CINAHL), and no current or ongoing protocols or reviews on the subject were identified. That said, the objective of this scoping review was to map available evidence on the experiences of adult patients and family members regarding the transition of care between intensive care and inpatient units.

## Method

### Type of study

This is a scoping review, guided by the JBI^(18)^. This review aimed to answer the guiding question: “What evidence is there in the literature on the experiences of adult patients and their families regarding the transition of care between the ICU and the inpatient unit?” This question was formulated using the acronym PCC, where P (population) refers to adult patients and/or family members, primary caregivers, or decision-makers; C (concept) refers to the experience of patients and/or family members; and C (context) refers to the transition of care between the ICU and the inpatient unit.

The protocol for this review was registered in the Open Science Framework (https://osf.io/72khs/), and this report was presented in accordance with the Preferred Reporting Items for Systematic Reviews and Meta-Analyses (PRISMA) extension for Scoping Reviews Checklist^(19)^.

### Eligibility criteria

Primary studies, reviews, dissertations, and theses were included that addressed the experience of adult patients or their family members undergoing care in an intensive care setting, regardless of the etiology of admission or length of stay, and who had been transferred to an inpatient unit, considering transfers between these different levels of care within the same institution, published in Portuguese, English, or Spanish. There was no restriction on inclusion by year of publication. Those that addressed transfer to palliative care, obstetric, or psychiatric units were excluded due to the unique characteristics of these patient profiles.

### Sources of information

To identify potentially relevant studies and descriptors, an initial search was conducted in the PubMed^®^ and CINAHL information sources. The words contained in the title and abstract of the selected studies, as well as the indexing terms used to describe them, were analyzed to develop a comprehensive strategy.

The final strategy, including the identified terms, was adapted to each listed information source, i.e., the PubMed^®^ and Virtual Health Library portals and the CINAHL, Cochrane Database of Systematic Reviews, Embase, Scopus, and Web of Science databases. A search was also conducted in the Brazilian Digital Library of Theses and Dissertations and the Networked Digital Library of Theses and Dissertations.

The initial search, as well as the search of records with the final strategy in the different sources of evidence, was conducted between August and September 2023, with an update conducted between September 3 and 9, 2024. Finally, the reference lists of the included studies were examined for additional sources not retrieved by the search strategy.

### Search strategy

The first search strategy was developed using Medical Subject Headings (MeSH) Terms Patient Discharge, Patient Participation, Patient-Centered Care, Continuity of Patient Care, Transitional Care, and Patient Transfer. The final strategy, in addition to the aforementioned descriptors, incorporated their respective synonyms and the MeSH Terms Transition in care, Critical Care, and Intensive Care Units, as well as the free terms Perception and Experience, combined using the Boolean operators OR and AND.

To elucidate the final strategy, Figure 1 shows the search for the PubMed^®^ portal. The search strategy adjusted for each information source can be found in the SciELO Data repository (https://doi.org/10.48331/scielodata.PDUSZX). It should be noted that the design and refinement of this outline relied on the collaboration of a librarian.

**Figure 1-:** Search strategy for the PubMed^®^ portal. Porto Alegre, RS, Brazil, 2024

**Database**	**Search strategy**
PubMed ^®^	(((((((((((((((((((“ *Patient Discharge* ”[MeSH *Terms* ]) *OR* (“ *Discharge, Patient* ”[MeSH *Terms* ])) *OR* (“ *Discharges* , *Patient* ”[MeSH *Terms* ])) *OR* (“ *Patient Discharges* ”[MeSH *Terms* ])) *OR* (“ *Discharge Planning* ”[MeSH *Terms* ])) *OR* (“ *Discharge Plannings* ”[MeSH *Terms* ])) *OR* (“ *Planning* , *Discharge* ”[MeSH *Terms* ])) *OR* (“ *Plannings* , *Discharge* ”[MeSH *Terms* ])) *OR* (“ *Patient Discharge* ”[ *Title* / *Abstract* ])) *OR* (“ *Patient Discharges* ”[ *Title* / *Abstract* ])) *OR* (“ *Discharge Planning* ”[ *Title* / *Abstract* ])) OR (“ *Discharge Plannings* ”[ *Title* / *Abstract* ])) *OR* (((((((((((((((((((((“ *Patient Participation* ”[MeSH *Terms* ]) *OR* (“ *Participation* , *Patient* ”[MeSH *Terms* ])) *OR* (“ *Patient* *Involvement* ”[MeSH *Terms* ])) *OR* (“ *Involvement* , *Patient* ”[MeSH *Terms* ])) *OR* (“ *Patient Empowerment* ”[MeSH *Terms* ])) *OR* (“ *Empowerment* , *Patient* ”[MeSH *Terms* ])) *OR* (“ *Patient Participation Rates* ”[MeSH *Terms* ])) *OR* (“ *Participation Rate* , *Patient* ”[MeSH *Terms* ])) *OR* (“ *Participation Rates* , *Patient* ”[MeSH *Terms* ])) *OR* (“ *Patient Participation Rate* ”[MeSH *Terms* ])) *OR* (“ *Patient Activation* ”[MeSH *Terms* ])) *OR* (“ *Activation* , *Patient* ”[MeSH *Terms* ])) *OR* (“ *Patient Engagement* ”[MeSH *Terms* ])) *OR* (“ *Engagement* , *Patient* ”[MeSH *Terms* ])) *OR* (“ *Patient Participation* ”[ *Title* / *Abstract* ])) *OR* (“ *Patient Involvement* ”[ *Title* / *Abstract* ])) *OR* (“ *Patient Empowerment* ”[ *Title* / *Abstract* ])) *OR* (“ *Patient Participation Rates* ”[ *Title* / *Abstract* ])) *OR* (“ *Patient Participation Rate* ”[ *Title* / *Abstract* ])) *OR* (“ *Patient Activation* ”[ *Title* / *Abstract* ])) *OR* (“ *Patient Engagement* ”[ *Title* / *Abstract* ]))) *OR* (((((((((((((((((((((((“ *Patient* - *Centered* *Care* ”[MeSH *Terms* ]) *OR* (“ *Care* , *Patient* - *Centered* ”[MeSH *Terms* ])) *OR* (“ *Patient Centered Care* ”[MeSH *Terms* ])) *OR* (“ *Person* - *Centered* *Care* ”[MeSH *Terms* ])) *OR* (“ *Care* , *Person* - *Centered* ”[MeSH *Terms* ])) *OR* (“ *Cares* , *Person* - *Centered* ”[MeSH *Terms* ])) *OR* (“ *Person* *Centered* *Care* ”[MeSH *Terms* ])) *OR* (“ *Patient* - *Focused Care* ”[MeSH *Terms* ])) *OR* (“ *Care* , *Patient* - *Focused* ”[MeSH *Terms* ])) *OR* (“ *Patient Focused Care* ”[MeSH *Terms* ])) *OR* (“ *Nursing* , *Patient* - *Centered* ”[MeSH *Terms* ])) *OR* (“ *Nursing* ,
PubMed ^®^	*Patient Centered* ”[MeSH *Terms* ])) *OR* (“ *Patient* - *Centered* *Nursing* ”[MeSH *Terms* ])) *OR* (“ *Patient* *Centered* *Nursing* ”[MeSH *Terms* ])) *OR* (“ *Patient* - *Centered* *Care* ”[ *Title* / *Abstract* ])) *OR* (“ *Patient Centered Care* ”[ *Title* / *Abstract* ])) *OR* (“ *Person* - *Centered* *Care* ”[ *Title* / *Abstract* ])) *OR* (“ *Person* *Centered* *Care* ”[ *Title* / *Abstract* ])) *OR* (“ *Person* - *Centered* *Cares* ”[ *Title* / *Abstract* ])) *OR* (“ *Patient* - *Focused* *Care* ”[ *Title* / *Abstract* ])) *OR* (“ *Patient Focused Care* ”[ *Title* / *Abstract* ])) *OR* (“ *Patient* - *Centered* *Nursing* ”[ *Title* / *Abstract* ])) *OR* (“ *Patient Centered Nursing* ”[ *Title* / *Abstract* ]))) *OR* (((((((((((((“ *Continuity of Patient Care* ”[MeSH *Terms* ]) *OR* (“ *Care Continuity* , *Patient* ”[MeSH *Terms* ])) *OR* (“ *Patient Care Continuity* ”[MeSH *Terms* ])) *OR* (“ *Continuum of Care* ”[MeSH *Terms* ])) *OR* (“ *Care Continuum* ”[MeSH *Terms* ])) *OR* (“ *Continuity of Care* ”[MeSH *Terms* ])) *OR* (“ *Care Continuity* ”[MeSH *Terms* ])) *OR* (“ *Continuity of Patient Care* ”[ *Title* / *Abstract* ])) *OR* (“ *Patient Care Continuity* ”[ *Title* / *Abstract* ])) *OR* (“ *Continuum of Care* ”[ *Title* / *Abstract* ])) *OR* (“ *Care Continuum* ”[ *Title* / *Abstract* ])) *OR* (“ *Continuity of Care* ”[ *Title* / *Abstract* ])) *OR* (“ *Care Continuity* ”[ *Title* / *Abstract* ]))) *OR* ((((((((((“ *Transitional Care* ”[MeSH *Terms* ]) *OR* (“ *Care* , *Transitional* ”[MeSH *Terms* ])) *OR* (“ *Cares* , *Transitional* ”[MeSH *Terms* ])) *OR* (“ *Transitional Cares* ”[MeSH *Terms* ])) *OR* (“ *Transition Care* ”[MeSH *Terms* ])) *OR* (“ *Transition Cares* ”[MeSH *Terms* ])) *OR* (“ *Transitional Care* ”[ *Title* / *Abstract* ])) *OR* (“ *Transitional Cares* ”[ *Title* / *Abstract* ])) *OR* (“ *Transition Care* ”[ *Title* / *Abstract* ])) *OR* (“ *Transition Cares* ”[ *Title* / *Abstract* ]))) *OR* (((((((((((((((((((((((((((((((((((((“ *Patient Transfer* ”[MeSH *Terms* ]) *OR* (“ *Patient Transfers* ”[MeSH *Terms* ])) *OR* (“ *Transfer* , *Patient* ”[MeSH *Terms* ])) *OR* (“ *Transfers* , *Patient* ”[MeSH *Terms* ])) *OR* (“ *Patient Transition* ”[MeSH *Terms* ])) *OR* (“ *Patient Transitions* ”[MeSH *Terms* ])) *OR* (“ *Transition* , *Patient* ”[MeSH *Terms* ])) *OR* (“ *Transitions* , *Patient* ”[MeSH *Terms* ])) *OR* (“ *Care Transition* ”[MeSH *Terms* ])) *OR* (“ *Care Transitions* ”[MeSH *Terms* ])) *OR* (“ *Transition* , *Care* ”[MeSH *Terms* ])) *OR* (“ *Transitions* , *Care* ”[MeSH *Terms* ])) *OR* (“ *Transition of Care* ”[MeSH *Terms* ])) *OR* (“ *Health Care Transition* ”[MeSH *Terms* ])) *OR* (“ *Care Transition* , *Health* ”[MeSH *Terms* ])) *OR* (“ *Care Transitions* , *Health* ”[MeSH *Terms* ])) *OR* (“ *Health Care Transitions* ”[MeSH *Terms* ])) *OR* (“ *Transition* , *Health Care* ”[MeSH *Terms* ])) *OR* (“ *Transitions* , *Health Care* ”[MeSH *Terms* ])) *OR* (“ *Patient Turfing* ”[MeSH *Terms* ])) *OR* (“ *Patient Turfings* ”[MeSH *Terms* ])) *OR* (“ *Turfing* , *Patient* ”[MeSH *Terms* ])) *OR* (“ *Turfings* , *Patient* ”[MeSH *Terms* ])) *OR* (“ *Patient Dumping* ”[MeSH *Terms* ])) *OR* (“ *Dumping* , *Patient* ”[MeSH *Terms* ])) *OR* (“ *Patient Transfer* ”[ *Title* / *Abstract* ])) *OR* (“ *Patient Transfers* ”[ *Title* / *Abstract* ])) *OR* (“ *Patient Transition* ”[ *Title* / *Abstract* ])) *OR* (“ *Patient Transitions* ”[ *Title* / *Abstract* ])) *OR* (“ *Care Transition* ”[ *Title* / *Abstract* ])) *OR* (“ *Care Transitions* ”[ *Title* / *Abstract* ])) *OR* (“ *Transition of Care* ”[ *Title* / *Abstract* ])) *OR* (“ *Health Care Transition* ”[ *Title* / *Abstract* ])) *OR* (“ *Health Care Transitions* ”[ *Title* / *Abstract* ])) *OR* (“ *Patient Turfing* ”[ *Title* / *Abstract* ])) *OR* (“ *Patient Turfings* ”[ *Title* / *Abstract* ])) *OR* (“ *Patient Dumping* ”[ *Title* / *Abstract* ]))) *OR* (“ *Transition in care* ”)) *AND* (((((((“ *Critical Care* ”[MeSH *Terms* ]) *OR* (“ *Care* , *Critical* ”[MeSH *Terms* ])) *OR* (“ *Intensive Care* ”[MeSH *Terms* ])) *OR* (“ *Care* , *Intensive* ”[MeSH *Terms* ])) *OR* (“ *Critical Care* ”[ *Title* / *Abstract* ])) *OR* (“ *Intensive Care* ”[ *Title* / *Abstract* ]) *OR* (((((((“ *Intensive Care Units* ”[MeSH *Terms* ]) *OR* (“ *Intensive Care Unit* ”[MeSH *Terms* ])) *OR* (“ *Unit* , *Intensive Care* ”[MeSH *Terms* ])) *OR* (“ *ICU Intensive Care Units* ”[MeSH *Terms* ])) *OR* (“ *Intensive Care Units* ”[ *Title* / *Abstract* ])) *OR* (“ *Intensive Care Unit* ”[ *Title* / *Abstract* ])) *OR* (“ *ICU Intensive Care Units* ”[ *Title* / *Abstract* ])))) *AND* (( *Perception* [ *Title* / *Abstract* ]) *OR* *(Experience* [ *Title* / *Abstract* ]))

### Selection of sources of evidence

The findings were uploaded to the EndNote Web reference manager for automatic removal of duplicates. Next, Rayyan software was used to continue this process, with manual exclusion of duplicate studies.

The titles and abstracts were evaluated by two reviewers in a blind and independent manner for selection according to the specified eligibility criteria. Any disagreements between the reviewers during the selection process were resolved by consensus. When agreement could not be reached, a third researcher was consulted for the final decision.

### Data extraction

The full text of the selected records was evaluated in detail by two independent researchers. A data extraction form was developed by the authors, containing the following items: author(s), title, journal, year, volume, issue, page(s), digital object identifier or access link, country where the study was conducted, type of publication, objective(s), inclusion and exclusion criteria, sample, data collection and analysis, main results, steps for the transition of care between intensive care and inpatient units, and evidence identified from the list of references.

Two researchers familiarized themselves with the form in a pilot test, i.e., at least two sources were consulted to ensure the form was suitable for the purpose of this investigation.

The data were extracted by one reviewer and a second researcher verified the information collected. Any disagreements between the researchers were evaluated by a third reviewer.

### Data processing and analysis

The summary of the extracted data was organized in the form of figures and descriptive analysis, aiming at alignment with the objective and research question. In addition, a basic qualitative content analysis, using a deductive approach^(20)^, was developed to categorize the experiences of patients and their families according to the stages of the transition of care between intensive care and the inpatient unit, namely: assessment, planning, execution, and follow-up^(8)^.

### Ethical aspects

As this is a review based on secondary data in the public domain and available in the literature, ethical assessment is not required. However, it should be noted that copyright has been respected with the appropriate citations and references from the studies consulted.

## Results

The database search identified 7,646 records, and another seven were considered potentially eligible through the reference lists of the selected sources, leaving 30 studies in the final sample, as shown in Figure 2.

**Figure 2-:**
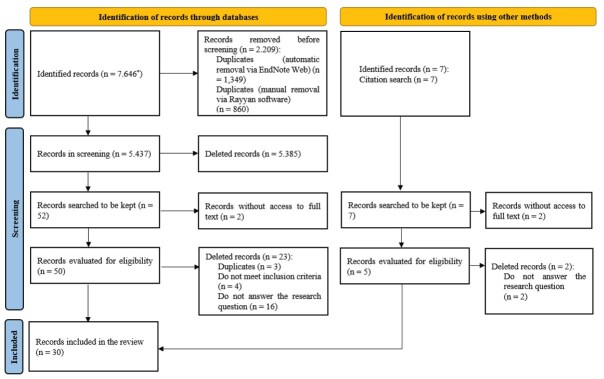
PRISMA Flowchart^(21)^. Porto Alegre, RS, Brazil, 2024

*PubMed^®^ (n = 1.328); *Biblioteca Virtual em Saúde* (n = 878); Cumulative Index to Nursing and Allied Health Literature (n = 1.374); Cochrane Database of Systematic Reviews (n = 138); Embase (n = 1.555); Scopus (n = 1.817); Web of Science (n = 505); *Biblioteca Digital Brasileira de Teses e Dissertações* (n = 13); Networked Digital Library of Theses and Dissertations (n = 38)

The characteristics of the records included, according to year, country, type of study, objective(s), and sample, are summarized in Figure 3.

**Figure 3-:** Characterization of the included studies. Porto Alegre, RS, Brazil, 2024

**Author(s), year**	**Country**	**Type of study**	**Objective(s)**	**Sample**
Leith, 1999 ^( 22 )^	Canada	Qualitative	Describe the perceptions of patients and their families regarding transfer from an ICU*.	Patients (n = 53) and family members (n = 35)
Odell, 2000 ^( 23 )^	England	Qualitative	Analyze how patients feel when transferred from the ICU* to the IU ^†^ after recovering from a critical illness or injury.	Patients (n = 6)
McKinney, et al., 2002 ^( 24 )^	Northern Ireland	Qualitative	Explore the experience of patients being transferred from the ICU* to the IU ^†^ .	Patients (n = 6)
Chaboyer, et al., 2005 ^( 25 )^	Australia	Qualitative	Explore the individual and collective perceptions of a group of patients and their families about transfer from the ICU*.	Patients (n = 7) and family members (n = 6)
Strahan, et al., 2005 ^( 26 )^	Northern Ireland	Qualitative	Explore and describe the experiences of patients after their transfer from the ICU*.	Patients (n = 10)
Field, et al., 2008 ^( 27 )^	England	Qualitative	Explore and examine the reports of former ICU* patients to identify additional causes of relocation stress.	Patients (n = 34)
Bench, et al., 2010 ^( 28 )^	England	Metasyntesis	Identify the most significant factors that impact the patient experience in the progress and recovery from a critical illness during the first month after discharge from the ICU*, and to discuss these factors in relation to the development of effective strategies to support discharge from the ICU*.	Records (n = 10) covering patients and family members
Bench, et al., 2011 ^( 29 )^	England	Qualitative	Seek the opinions of patients, family members, and healthcare professionals on the most effective methods of providing information about discharge from the ICU*, the content of the information needed, the benefits and limitations of existing information, and the potential implications in terms of resources.	Patients (n = 11), family members (n = 8), and healthcare professionals (n = 23)
Forsberg. et al., 2011 ^( 30 )^	Sweden	Qualitative	Describe the experiences of people who received care in the ICU* and were transferred to an IU ^†^ .	Patients (n = 10)
Calatayud, et al., 2013 ^( 31 )^	Spain	Literature review	Identify, explore, and critically present existing evidence on how patients, family members, and nursing professionals experience the transition process from the ICU* to the IU ^†^ , and analyze possible existing interventions for the development of an optimal transition process.	Records (n = 23) covering patients and family members
Cullinane, et al., 2013 ^( 32 )^	England	Literature review	Analyze the evidence on the transfer of patients from the ICU* to the IU ^†^ .	Records (n = 8) covering patients and family members
Cypress, 2013 ^( 33 )^	United States	Literature review	Systematically review the effects of transfer or discharge from the ICU* to medical-surgical inpatient units on critically ill adult patients, their families, and nurses.	Records (n = 27) covering patients, family members, and healthcare professionals
Häggström, et al., 2014 ^( 34 )^	Sweden	Mixed methods	Investigate family members’ perceptions of the quality of care during the process of transferring a patient from an ICU* to an IU ^†^ .	Family members (n = 65)
Ramsay, et al., 2014 ^( 35 )^	Scotland	Mixed methods	Explore the psychosocial needs of patients discharged from the ICU*, as captured using transition theory and the potential development of the role of proximity, follow-up, and intensive care liaison services.	Patients (n = 20)
Gill, et al., 2016 ^( 36 )^	Canada	Qualitative	Explore the experiences and perspectives of patients and their families on ICU* care without undue influence from caregivers or “traditional” researchers.	Patients and family members (n = 32)
Stelfox, et al., 2017 ^( 37 )^	Canada	Qualitative	Understand current transfer procedures and the experiences of patients and professionals (doctors and nurses in the ICU ^*^ and IU ^†^ ), from a 360-degree perspective, regarding transfers from the ICU* to the IU ^†^ , in order to inform recommendations for improving care.	Patients (n = 451)
Antonio, et al., 2018 ^( 38 )^	Brazil	Qualitative	Understanding, from the perspective of caregivers, the transition of patients discharged from ICUs*.	Family members (n = 30)
de Grood, et al., 2018 ^( 39 )^	Canada	Qualitative	Assess patients’ and caregivers’ perspectives on barriers and facilitators to high-quality transfers and recommendations for improving the transfer process.	Patients, family members, and healthcare professionals (n = 35)
Koilor, et al., 2018 ^( 40 )^	United States	Qualitative	Characterize the initial family experience of recovery from critical traumatic illness.	Family members (n = 13)
King, et al., 2019 ^( 41 )^	Canada	Scoping review	Describe what types of support patients need after discharge from the ICU* and how support needs differ across the recovery continuum (from ICU* discharge to long-term community-based recovery).	Records (n = 32) covering patients
Herling, et al., 2020 ^( 42 )^	Denmark	Qualitative	Explore the experiences of ICU* patients and their families during the transition to the IU ^†^ in order to find ways to support them during the transition process.	Patients (n = 10) and family members (n = 4)
Op ’t Hoog, et al., 2020 ^( 43 )^	Netherlands	Qualitative	Gain a deeper understanding of the experiences and needs of ICU* patients’ families during the transition from the ICU* to an IU ^†^ .	Family members (n = 13)
Ghorbanzadeh, et al., 2021 ^( 44 )^	Iran	Qualitative	Investigate the understanding and challenges patients face during their transition process from the ICU*.	Patients and family members (n = 8) and healthcare professionals (n = 14)
Lee, et al., 2021 ^( 45 )^	South Korea	Qualitative	Gain an in-depth understanding of the ICU* patient transfer experience in South Korea through a phenomenological analysis.	Patients (n = 15)
Cuzco, et al., 2022 ^( 46 )^	Spain	Qualitative	Describe patients’ experiences during the transition from the ICU* to the IU ^†^ .	Patients (n = 48)
Zhan, et al., 2022 ^( 47 )^	China	Qualitative	Explore the experiences and needs of family caregivers in relation to transitional care during the transfer from an ICU* to a IU ^†^ .	Family members (n = 15)
Cuzco, et al., 2023 ^( 48 )^	Spain	Qualitative	Explore the characteristics of the transition from the ICU* to the IU ^†^ based on the patient’s experience, and identify nursing therapy during the transfer of patients from the ICU* to the IU ^†^ .	Patients (n = 48)
Gullberg, et al., 2023 ^( 49 )^	Sweden	Qualitative	Describe patients’ experiences in preparing for transfer from an ICU* to an IU ^†^ .	Patients (n = 14)
Pastor, et al., 2023 ^( 50 )^	Spain	Qualitative	Understand the feelings and emotions that arise from the experience of discharge from an ICU*, and identify the factors that trigger well-being and emotional discomfort in patients.	Patients (n = 20)
Meiring- Noordstra, et al., 2024 ^( 51 )^	Netherlands	Qualitative	Explore the experiences of family members of patients admitted to the ICU* with acute conditions in the transition from the ICU* to an IU ^†^ and then home.	Family members (n = 12)

^*^ICU = Intensive Care Unit; ^†^IU = Inpatient Unit

Publications from 1999 to 2024 were identified, with emphasis on the years 2023 (n = 4), 2018 (n = 3), and 2013 (n = 3). In terms of origin, studies conducted by researchers from the United Kingdom (n = 7), Canada (n = 5), and Spain (n = 4) were highlighted. Regarding the type of study, the authors mainly developed qualitative primary research (n = 21) to address the theme, considering patients (n = 13) as the prevalent population of interest, followed by the dyad (patients and family members) (n = 7) and family members only (n = 6). The included studies were predominantly conducted by authors with backgrounds in nursing and medicine, including healthcare professionals, managers, teachers, and researchers.

Data collection procedures for the included studies were performed at different times: in the inpatient unit after discharge from the ICU, ranging from one to seven days after transfer^(23-24,26,37,40,42-43,45-48)^; and at home, by telephone, video call, or upon return to the institution as agreed, for months^(25,28,30,35,40,42,49,51)^ and years^(27,29,36)^ after discharge from the hospital.

The experiences of patients and their families, as described in the mapped publications and categorized according to the stages of transition from intensive care to inpatient care, are shown in Figure 2 Figure 4. A complete overview of the experiences is available in the SciELO Data repository (https://doi.org/10.48331/scielodata.PDUSZX).

**Figure 2-:**
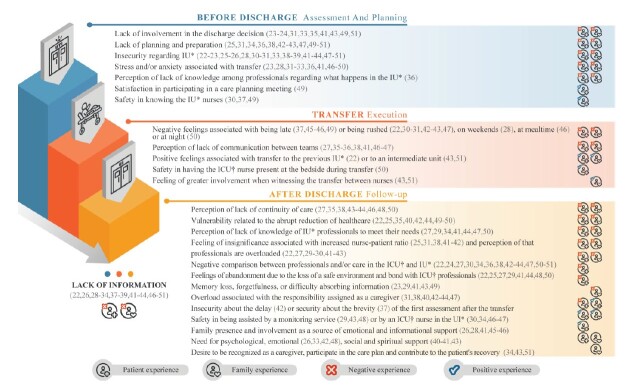
Figure 4– Experiences of patients and family members in the transition of care between intensive care and inpatient units. Porto Alegre, RS, Brazil, 2025

^*^IU = Inpatient Unit; ^†^ICU = Intensive Care Unit

## Discussion

The experiences of patients and their families were categorized in an innovative way, in light of the stages described for the transition of care between intensive care and inpatient units: discharge assessment, discharge planning, discharge execution, and post-discharge follow-up^(8)^. This analytical framework allowed us to identify critical and recurring points in the process, highlighting the transition as a sensitive phase of hospitalization, marked by high emotional stress and information gaps across all stages. The lack of adequate preparation for discharge, combined with deficiencies in communication between units, are factors that hinder continuity of care and individuals’ adaptation to the new environment.

The findings of this review contribute to the systematization of evidence that gives voice to the individuals who experience the transition, revealing a persistent scenario of weaknesses that have yet to be overcome: over more than two decades, negative experiences of patients and family members have predominated in this context. In addition, they point to promising directions for future research, aimed at deepening the understanding of the specific demands of individuals, as well as assessing the impact of their experiences on recovery and continuity of care.

Patients and family members experience a situational and organizational transition. Situational, because they experience a health-illness transition from admission, critical diagnosis, recovery, and discharge from intensive care, and organizational, as they move between two units with different configurations, inherent to their respective levels of care. This transition, in itself, exposes the individual to a situation of vulnerability^(1,52)^.

In the discharge assessment stage, there is a growing movement among professionals toward establishing objective criteria for this decision, based on the individual’s organic stability and the capacity of the receiving unit. However, the views of the patient and family are rarely discussed^(12,53-54)^, corroborating the manifestations of lack of involvement and detachment regarding the decision made by the professional. Unlike the context of hospital discharge, which already has a validated instrument translated into several languages to assess the patient’s perception of readiness^(55-56)^.

Critical patients often lose their autonomy and find themselves dependent on others for basic activities of daily living. Knowledge and preparation for change facilitate the empowerment of individuals to adapt to their new circumstances and regain their independence. Health education is one of the main nursing interventions in this transition process^(57-58)^. Work involving the development of a model^(59)^ or a package of measures^(60)^ for the transition of care upon discharge from the ICU consider information to be a facilitator of this process, since it empowers, provides tools for active participation, and promotes collaborative relationships.

The gradual sharing of information, repeated and supported by written material and listening spaces for clarifying doubts, conducted in a respectful manner, are the foundations of successful transitions^(8,61-62)^. Initiatives to involve and empower individuals are reported for ICU discharge^(63-64)^, family-centered education^(65)^, and care after this critical moment^(66)^. However, in line with the results found in this review, other studies indicate that patients and family members are often excluded from discharge planning, receiving insufficient and conflicting information, with technical terms and little time devoted to this activity^(6,8)^.

The ideal time frame for this planning is still unknown, but periods of less than six hours and more than 48 hours have been considered by intensive care professionals and managers^(12)^. However, pressure for bed availability^(67-68)^, combined with high workloads^(69)^, often create such a turbulent environment that instructional opportunities are often overlooked^(6,69)^.

Communication barriers and inadequate preparation are factors related to the nursing diagnosis “relocation stress syndrome,” defined as a physiological and/or psychosocial disturbance associated with transfer from one environment to another, characterized, among other things, by concern, insecurity, anxiety, and fear^(70)^ – feelings consistently mentioned in the selected sample. Researchers validated a self-report tool to assess relocation stress in patients after transfer from the ICU, with the purpose of identifying psychological problems during the move and assisting professionals in developing person-centered care, aiming to minimize the disturbances associated with the transition period^(71)^.

The transfer of bedside care for discharge^(69,72)^ was identified as a positive experience by patients and family members and has been reported as a favorable strategy for the transition of care. On the other hand, delays due to the unavailability of beds in the inpatient unit^(73-75)^, transfers on weekends^(75-77)^, during shift changes^(77)^, or at night^(77-78)^ contributed to individual dissatisfaction and have been associated with negative outcomes.

Regarding communication between ICU and inpatient unit teams, both professionals^(79)^ and patients and family members pointed out weaknesses in this process. Studies show that, despite guidelines, communication lacks standardization and, commonly, the data transmitted is at the discretion of ICU professionals^(8,80-82)^. The literature also emphasizes that current practices fall short of the ideal, with discrepancies between the information transmitted by the intensive care team and the needs of those who receive it in the inpatient unit^(8,69,77,80,82)^.

ICU professionals often overestimate the capacity of the receiving team^(13-14,79,82)^, which, in turn, perceives this transition as a great responsibility. This perception is intensified by the arrival of unstable patients with complex histories, unclear treatments, transferred prematurely, and insufficiently prepared to leave the safe environment of intensive care. The challenge becomes even greater when the receiving team feels inexperienced or lacks the qualifications to care for this type of patient^(67,69)^, a weakness perceived by both patients and family members. Nurses from inpatient units suggested as improvement strategies prior contact with intensive care nurses to exchange information and the possibility of spending a period in the ICU to observe the procedures performed and deepen their understanding of critical care^(67)^. This practice also benefits patients and family members by providing an opportunity for initial contact with the receiving team’s professionals before the transfer, promoting continuity and humanization of care^(83)^.

Among the publications analyzed, the abrupt and poorly communicated reduction in care stood out as a stressful experience. When there is no guidance on the expected differences in the intensity of support between units, the individual’s perception can be distorted, leading to unrealistic comparisons. In addition, there is a mismatch between the dependence on intensive care received in the ICU and the need for self-sufficiency to perform the same tasks in the inpatient unit, without sufficient time for this adaptation. Family members often report adversities related to discharge from intensive care, such as loss of information, reduced monitoring, and the feeling of premature transfer^(11)^. To improve the quality of the transition and ensure patient safety, it is essential to implement practices such as gradually reducing the level of care while still in the ICU, including the removal of unnecessary invasive devices, the progressive withdrawal of multiparametric monitoring, and the assessment of mobility^(6,84-85)^.

Furthermore, the presence and involvement of the family are essential to the ICU discharge process. Many individuals experience discharge from intensive care with altered levels and/or content of consciousness, with significant cognitive and/or communication deficits, or dependence for basic activities of daily living, and family members act as a link for emotional and informational support, assisting their adaptation to the new phase of recovery and, in many cases, assuming decision-making roles regarding conduct and treatment^(6,8,86)^ They actively seek to be recognized as an essential part of their family member’s care^(87)^.

Follow-up programs after discharge from the ICU have been implemented in various ways. Care transition teams monitor the patient in the inpatient unit for at least 24 hours after discharge from intensive care^(88-90)^, while liaison nursing services perform activities focused on safety, airway management, infection and fall prevention, skin care, and education and support for patients and family members^(91-92)^. Although systematic reviews and meta-analyses indicate limited evidence on the effectiveness of these interventions in reducing readmissions and mortality^(93-94)^, family members reported that their needs were met and consider the work of liaison nurses valuable, especially for the support offered during the transition^(95)^.

One study highlighted the benefits of a Post-Intensive Care Group, in which ICU nurses provided additional care to the most vulnerable patients in the inpatient unit. This care was tailored to the physical and psychological needs of patients, including more frequent visits when necessary, which promoted greater confidence and reduced anxiety among both patients and their families. The group’s main activities focused on prevention, with early detection of signs of deterioration, mitigation of adverse events, and promotion of knowledge exchange among teams, with a focus on the patient’s best interests^(96)^.

In addition, the 30 studies analyzed were published in a balanced manner over time and conducted by different professional profiles, working in care, management, teaching, and research. This distribution highlights a constant demand for knowledge production on the topic, as well as a shared interest among different segments of practice and science in understanding and qualifying this process. Although the predominance falls on authors from nursing and medicine, the complexity of the phenomenon investigated requires integrated approaches and the development of future studies that incorporate the perspective of the multidisciplinary team.

More than half of the studies were conducted in Europe, reflecting a concentration of scientific production in a few countries and highlighting the need to explore this topic in other contexts. Scope reviews that addressed the transition of care between the ICU and inpatient units for adult^(6)^ and neonatal, pediatric, and adult^(8)^ patients also pointed to Europe and North America as the main sources of most of the publications included.

Another relevant topic regarding the characteristics of the studies is the variability in the data collection periods adopted by the researchers. There is no description of the ideal time interval; it seems that authors consider key periods to capture impressions of contextual interest. For example, to understand how patients and their families experience the intensive care environment and its impact on recovery, data were collected in the first week after discharge from the ICU^(97)^, while to investigate the effects of virtual reality on mental health, perceived quality, and satisfaction after intensive treatment among COVID-19 survivors, information was collected up to six months after hospital discharge^(98)^.

Among the limitations of this scoping review are language restrictions and the inaccessibility of the full text of four records, which may have resulted in an incomplete and less representative sample. Similarly, although data extraction was performed systematically by two reviewers, it may not have been entirely accurate and exhaustive.

This review contributes to the advancement of scientific knowledge in the field of health and nursing by gathering evidence from different contexts and time periods and increasing the visibility of the stages of the transition of care between intensive care and inpatient units from the perspective of patients and family members. The representation created (Figure 4) can support continuing education activities in health by synthesizing and operationalizing the available knowledge, with an emphasis on the weaknesses identified at each stage of this process. The findings imply the need to improve the strategies currently adopted through structured and timely interventions, with a view to improving continuity of care and reducing the negative experiences that are still recurrent throughout this trajectory.

## Conclusion

The transition of care between intensive care and inpatient units is a complex and multifaceted process, often associated with challenges that affect patients, family members, and healthcare teams. The experiences described in the mapped publications highlight this moment as a significant event in hospitalization, often accompanied by emotional impact. A lack of information was identified at all stages of the process, with recurring reports of unpreparedness and perceptions of abandonment, especially in cases of sudden transfer. In addition, inadequate communication between the parties involved resulted in gaps in care, hindering individuals’ adaptation and compromising the transition.

In this context, health education stands out as one of the main nursing interventions to reduce the gaps identified. Providing clear and timely information, explaining the expected differences between levels of care, and adopting structured communication are key strategies. This review confirms the categorization of patient and family experiences between the stages of transition from intensive care to inpatient care as a relevant contribution, providing a clear and accessible overview of the main critical points in the process. This systematization can be used as a strategic resource in continuing health education activities, promoting the translation of knowledge into practice and indicating aspects that require attention at each stage.

Furthermore, it is essential to promote an organizational culture with clearly defined responsibilities among the professional categories involved in preparing the patient and family. This approach should replace isolated practices and minimize reliance on subjective actions, strengthening a collaborative care environment.

Advancing understanding of this topic requires the development of evidence-based strategies, including integrated transition models with standardized communication, educational programs to prepare patients and family members before transfer, and validated instruments to measure perceptions of preparation, satisfaction, and safety during the process. Future research can contribute to identifying practices that promote autonomy, empowerment, and safety and to better understanding the impact of care transition on lived experiences. In this way, it will be possible not only to reduce the stress and vulnerability associated with this moment but also to ensure equitable, efficient, and person-centered care.

## Data Availability

The dataset of this article is available on the RLAE page in the SciELO Data repository, at the link https://doi.org/10.48331/scielodata.PDUSZX
