# Supplementary material for: Transition experiences of patients and families upon discharge from intensive care: a scoping review
Source: Rev Lat Am Enfermagem. 2025 Dec 1;33:e4762. doi: 10.1590/1518-8345.7919.4762 (PMC12685361; doi:10.1590/1518-8345.7919.4762)
Supplement: Translation

# Translation: Experiências de transição de pacientes e familiares na alta da terapia intensiva: revisão de escopo*

**DOI:** 10.1590/1518-8345.7919.4763

**Authors:** Paula Buchs Zucatti, Clediane Rita Portalupi da Trindade, Rosana Evangelista-Poderoso, Edinêis de Brito Guirardello, Maria Alice Dias da Silva Lima

**Affiliations:** 1 Universidade Federal do Rio Grande do Sul, Escola de Enfermagem e de Saúde Coletiva, Porto Alegre, RS, Brasil. Universidade Federal do Rio Grande do Sul Escola de Enfermagem e de Saúde Coletiva RS Porto Alegre Brasil; 2 Hospital de Clínicas de Porto Alegre, Centro de Tratamento Intensivo, Porto Alegre, RS, Brasil. Hospital de Clínicas de Porto Alegre Centro de Tratamento Intensivo RS Porto Alegre Brasil; 3 Universidade Estadual de Campinas, Biblioteca da Faculdade de Ciências Médicas, Campinas, SP, Brasil. Universidade Estadual de Campinas Biblioteca da Faculdade de Ciências Médicas SP Campinas Brasil; 4 Universidade Estadual de Campinas, Faculdade de Enfermagem, Campinas, SP, Brasil. Universidade Estadual de Campinas Faculdade de Enfermagem SP Campinas Brasil; 5 Bolsista do Conselho Nacional de Desenvolvimento Científico e Tecnológico (CNPq), Brasil. Bolsista do Conselho Nacional de Desenvolvimento Científico e Tecnológico Brasil

**Keywords:** Alta do Paciente, Assistência Centrada no Paciente, Continuidade da Assistência ao Paciente, Cuidado Transicional, Participação do Paciente, Transferência de Pacientes

## Abstract

mapear evidências disponíveis sobre as experiências de pacientes adultos e familiares em relação à transição do cuidado entre terapia intensiva e unidade de internação.

revisão de escopo conduzida conforme diretrizes do *Joanna BriggsInstitute*. Foram incluídos estudos primários, revisões, dissertações e teses, publicados em português, inglês ou espanhol, sem limite temporal. A busca final ocorreu em portais e bases de dados em saúde e bibliotecas digitais. A seleção foi realizada por duas revisoras de forma cega e independente. Os dados foram extraídos por meio de roteiro e apresentados em figuras, análise descritiva e qualitativa dedutiva, categorizando as vivências entre as etapas de avaliação, planejamento, execução e acompanhamento após a alta.

trinta artigos compuseram a amostra. As experiências mapeadas evidenciam a transição como um momento crítico da hospitalização, marcado por impacto emocional e fragilidades informacionais. Relatos de despreparo, sensação de abandono e falhas de comunicação comprometem a continuidade do cuidado e a adaptação de pacientes e familiares.

as experiências descritas apontam a transição do cuidado entre terapia intensiva e unidade de internação como processo complexo e multifacetado. As lacunas identificadas demandam estratégias de comunicação estruturada, informações oportunas e alinhamento entre as unidades.

## Destaques

**(1)** Transição entre terapia intensiva e unidade de internação é complexa e desafiadora. **(2)** Pacientes e familiares experienciam redução abrupta e descontinuidade do cuidado. **(3)** Comunicação imprecisa ou insuficiente está presente em todas as etapas do processo. **(4)** Presença e envolvimento da família como elo de suporte emocional e informacional.

## Introdução

A alta da Unidade de Terapia Intensiva (UTI) para a unidade de internação representa uma mudança significativa, marcando um processo de transição importante para o paciente e sua família. Enquanto a mudança é um evento específico e delimitado no tempo, a transição é um processo contínuo, caracterizado por uma série de respostas. Durante esse período, os indivíduos enfrentam contextos, sensações e emoções desconhecidas, além de lidarem com incertezas sobre o futuro, o que os coloca em uma condição de vulnerabilidade^(1)^.

A forma como a pessoa interpreta a mudança, seu significado e a disponibilidade de apoio e de recursos repercutem diretamente nas suas experiências, podendo favorecer ou dificultar a sua recuperação^(1)^. A experiência do paciente resulta da soma de todas as interações moldadas pela cultura organizacional, que influenciam suas percepções ao longo do *continuum* de cuidados^(2)^.

A preparação dos pacientes e seus familiares para lidarem com a transição entre os diferentes níveis assistenciais, bem como a garantia da continuidade dos cuidados até que as mudanças sejam incorporadas ao seu cotidiano desempenham papel fundamental. Essa experiência abrange sua compreensão sobre o processo de alta, seu nível de conhecimento e de engajamento, as ações dos profissionais que condicionam as vivências e a concretização ou não de suas expectativas^(1)^.

Além da fragilidade situacional do indivíduo, a transferência de cuidados intensivos para cuidados regulares é reconhecida como um ponto crítico no decorrer da internação hospitalar. Este processo é especialmente suscetível a falhas, seja no deslocamento do paciente, seja na transferência das informações entre os setores do sistema de saúde. Nesse sentido, a transição adequada do cuidado, compreendida como um conjunto estruturado de ações realizadas pelos profissionais de saúde para assegurar a coordenação e a continuidade dos cuidados, faz-se essencial^(3-5)^.

No cenário de alta da UTI, a fragmentação do cuidado tem sido associada a desfechos negativos, como aumento do estresse e da insatisfação de pacientes e familiares, maior tempo de internação, eventos adversos, elevação dos custos hospitalares, readmissões e óbitos^(6-10)^. Ademais, a experiência dos indivíduos na transição entre terapia intensiva e unidade de internação é influenciada pelo planejamento, pela comunicação e pela execução desse processo^(11)^.

Uma pesquisa recente com profissionais e gestores de terapia intensiva revelou lacunas entre o momento em que o paciente deixa de necessitar de cuidados intensivos e aquele em que pode realizar de forma segura a transição para os cuidados regulares^(12)^. Esta discrepância reforça a percepção, relatada por enfermeiros de unidades de internação, de que existem “dois mundos distintos”: enquanto os profissionais de terapia intensiva têm expectativas elevadas sobre as capacidades de manejo da equipe de internação, essas demandas podem representar uma ameaça à segurança do paciente^(13-14)^. Além disso, os próprios pacientes descrevem dificuldades ao longo de sua recuperação, destacando a falta de informações claras e acessíveis, bem como a ausência de espaços para a escuta e o esclarecimento de dúvidas, o que compromete a experiência de cuidado durante esse período crítico^(15-17)^.

Uma abordagem estruturada envolve etapas sequenciais para a transição do cuidado entre terapia intensiva e unidade de internação. Essas etapas incluem: 1) *avaliação para a alta* com a utilização de escores específicos para determinar gravidade, riscos e evolução clínica; 2) *planejamento da alta,* que consiste na sumarização dos problemas de saúde, na revisão das metas de atendimento, na reconciliação medicamentosa e na orientação detalhada ao paciente e à família sobre os cuidados recebidos, os planejados, o processo de alta e o local de destino; 3) *execução da alta*, que envolve a decisão sobre o momento oportuno para a transferência, a identificação da equipe receptora e a comunicação efetiva entre as partes envolvidas; e 4) *acompanhamento após a alta*, com a prestação da assistência ao paciente na unidade de internação por um membro da equipe da UTI, assegurando a continuidade do cuidado e facilitando a adaptação ao novo ambiente^(8)^.

Embora as experiências dos pacientes e seus familiares no contexto da alta da UTI tenham sido amplamente discutidas na literatura ao longo dos últimos anos^(6,8)^, abordá-las com base nas etapas para a transição do cuidado entre terapia intensiva e unidade de internação constitui uma perspectiva inovadora. O enfoque permite identificar pontos críticos do processo e determinar o momento mais adequado para intervenções específicas, contribuindo para aprimorar a qualidade das transições e mitigar os impactos negativos dessa experiência no período imediato após a alta.

A partir do exposto, foi realizada uma busca preliminar no *Joanna BriggsInstitute* (JBI) *Evidence Synthesis*, no PubMed® e no *Cumulative Index to Nursing and Allied Health Literature* (CINAHL) e não foram identificados protocolos ou revisões atuais ou em andamento sobre a temática. Posto isto, o objetivo desta revisão de escopo foi mapear evidências disponíveis sobre as experiências de pacientes adultos e familiares em relação à transição do cuidado entre terapia intensiva e unidade de internação.

## Método

### Tipo do estudo

Trata-se de uma revisão de escopo, orientada pelo JBI^(18)^. Esta revisão objetivou responder à questão norteadora: “Quais as evidências na literatura sobre as experiências dos pacientes adultos e seus familiares em relação à transição do cuidado entre UTI e unidade de internação?”. A elaboração da pergunta foi feita por meio da utilização do acrônimo PCC, sendo P (população) – pacientes adultos e/ou familiares – membro da família, cuidador principal ou responsável pela tomada de decisão; C (conceito) – experiência do paciente e/ou dos familiares; e C (contexto) – transição do cuidado entre UTI e unidade de internação.

O protocolo desta revisão foi registrado na *Open Science Framework* (https://osf.io/72khs/), e este relatório, apresentado em consonância com o disposto no *Preferred Reporting Items for Systematic reviews and Meta-Analyses* (PRISMA) *extension for Scoping Reviews Checklist*^(19)^.

### Critérios de elegibilidade

Foram incluídos estudos primários, revisões, dissertações e teses, que abordassem a experiência de pacientes adultos, ou de seus familiares, submetidos a cuidados em um ambiente de terapia intensiva, independentemente da etiologia da admissão ou do período de internação, e que tivessem sido transferidos para uma unidade de internação, considerando-se a transferência entre os diferentes níveis assistenciais dentro de uma mesma instituição, publicados em português, inglês ou espanhol. Não houve restrição quanto à inclusão por ano de publicação. Foram excluídos aqueles que abordassem a transferência para unidades de cuidados paliativos, obstétricos ou psiquiátricos, devido às singularidades desses perfis de pacientes.

### Fontes de informação

Para a identificação de estudos e descritores potencialmente relevantes, fez-se uma busca inicial nas fontes de informação PubMed^®^ e CINAHL. As palavras contidas no título e no resumo dos trabalhos selecionados, assim como os termos de indexação usados para descrevê-los, foram analisados para desenvolver uma estratégia completa.

A estratégia final, incluindo os termos identificados, foi adaptada para cada fonte de informação elencada, ou seja, os portais PubMed^®^ e Biblioteca Virtual em Saúde e as bases de dados CINAHL, Cochrane *Database of Systematic Reviews*, Embase, Scopus e *Web of Science*. Também foi realizada uma busca na Biblioteca Digital Brasileira de Teses e Dissertações e na *Networked Digital Library of Theses and Dissertations*.

A busca inicial, bem como a pesquisa dos registros com a estratégia final nas diferentes fontes de evidência, foi realizada entre agosto e setembro de 2023, com atualização conduzida no período de três a nove de setembro de 2024. Por fim, as listas de referências dos estudos incluídos foram examinadas em busca de fontes adicionais não recuperadas pela estratégia de busca.

### Estratégia de busca

A primeira estratégia de busca foi desenvolvida utilizando-se os *Medical Subject Headings* (MeSH) *TermsPatient Discharge*, *Patient Participation*, *Patient-Centered Care*, *Continuity of Patient Care*, *Transitional Care* e *Patient Transfer*. A estratégia final, além dos descritores mencionados, incorporou seus respectivos sinônimos e os MeSH *Terms Transitionin care*, *Critical Care* e *Intensive Care Units*, bem como os termos livres *Perception* e *Experience*, combinados por meio dos operadores booleanos *OR* e *AND*.

Para elucidar a estratégia final, apresenta-se na Figura 1, a busca para o portal PubMed^®^. A estratégia de busca ajustada para cada fonte de informação pode ser encontrada no repositório de dados SciELO Data (https://doi.org/10.48331/scielodata.PDUSZX). Destaca-se que a concepção e o refinamento desse delineamento contaram com a colaboração de uma bibliotecária.

**Figura 1-:** Estratégia de busca para o portal PubMed^®^. Porto Alegre, RS, Brasil, 2024

**Fonte de informação**	**Estratégia de busca**
PubMed ^®^	(((((((((((((((((((“ *Patient Discharge* ”[MeSH *Terms* ]) *OR* (“ *Discharge, Patient* ”[MeSH *Terms* ])) *OR* (“ *Discharges* , *Patient* ”[MeSH *Terms* ])) *OR* (“ *Patient Discharges* ”[MeSH *Terms* ])) *OR* (“ *Discharge Planning* ”[MeSH *Terms* ])) *OR* (“ *Discharge Plannings* ”[MeSH *Terms* ])) *OR* (“ *Planning* , *Discharge* ”[MeSH *Terms* ])) *OR* (“ *Plannings* , *Discharge* ”[MeSH *Terms* ])) *OR* (“ *Patient Discharge* ”[ *Title* / *Abstract* ])) *OR* (“ *Patient Discharges* ”[ *Title* / *Abstract* ])) *OR* (“ *Discharge Planning* ”[ *Title* / *Abstract* ])) OR (“ *Discharge Plannings* ”[ *Title* / *Abstract* ])) *OR* (((((((((((((((((((((“ *Patient Participation* ”[MeSH *Terms* ]) *OR* (“ *Participation* , *Patient* ”[MeSH *Terms* ])) *OR* (“ *Patient* *Involvement* ”[MeSH *Terms* ])) *OR* (“ *Involvement* , *Patient* ”[MeSH *Terms* ])) *OR* (“ *Patient*
PubMed ^®^	*Empowerment* ”[MeSH *Terms* ])) *OR* (“ *Empowerment* , *Patient* ”[MeSH *Terms* ])) *OR* (“ *Patient Participation Rates* ”[MeSH *Terms* ])) *OR* (“ *Participation Rate* , *Patient* ”[MeSH *Terms* ])) *OR* (“ *Participation Rates* , *Patient* ”[MeSH *Terms* ])) *OR* (“ *Patient Participation Rate* ”[MeSH *Terms* ])) *OR* (“ *Patient Activation* ”[MeSH *Terms* ])) *OR* (“ *Activation* , *Patient* ”[MeSH *Terms* ])) *OR* (“ *Patient Engagement* ”[MeSH *Terms* ])) *OR* (“ *Engagement* , *Patient* ”[MeSH *Terms* ])) *OR* (“ *Patient Participation* ”[ *Title* / *Abstract* ])) *OR* (“ *Patient Involvement* ”[ *Title* / *Abstract* ])) *OR* (“ *Patient Empowerment* ”[ *Title* / *Abstract* ])) *OR* (“ *Patient Participation Rates* ”[ *Title* / *Abstract* ])) *OR* (“ *Patient Participation Rate* ”[ *Title* / *Abstract* ])) *OR* (“ *Patient Activation* ”[ *Title* / *Abstract* ])) *OR* (“ *Patient Engagement* ”[ *Title* / *Abstract* ]))) *OR* (((((((((((((((((((((((“ *Patient* - *Centered* *Care* ”[MeSH *Terms* ]) *OR* (“ *Care* , *Patient* - *Centered* ”[MeSH *Terms* ])) *OR* (“ *Patient Centered Care* ”[MeSH *Terms* ])) *OR* (“ *Person* - *Centered* *Care* ”[MeSH *Terms* ])) *OR* (“ *Care* , *Person* - *Centered* ”[MeSH *Terms* ])) *OR* (“ *Cares* , *Person* - *Centered* ”[MeSH *Terms* ])) *OR* (“ *Person* *Centered* *Care* ”[MeSH *Terms* ])) *OR* (“ *Patient* - *Focused Care* ”[MeSH *Terms* ])) *OR* (“ *Care* , *Patient* - *Focused* ”[MeSH *Terms* ])) *OR* (“ *Patient Focused Care* ”[MeSH *Terms* ])) *OR* (“ *Nursing* , *Patient* - *Centered* ”[MeSH *Terms* ])) *OR* (“ *Nursing* , *Patient Centered* ”[MeSH *Terms* ])) *OR* (“ *Patient* - *Centered* *Nursing* ”[MeSH *Terms* ])) *OR* (“ *Patient* *Centered* *Nursing* ”[MeSH *Terms* ])) *OR* (“ *Patient* - *Centered* *Care* ”[ *Title* / *Abstract* ])) *OR* (“ *Patient Centered Care* ”[ *Title* / *Abstract* ])) *OR* (“ *Person* - *Centered* *Care* ”[ *Title* / *Abstract* ])) *OR* (“ *Person* *Centered* *Care* ”[ *Title* / *Abstract* ])) *OR* (“ *Person* - *Centered* *Cares* ”[ *Title* / *Abstract* ])) *OR* (“ *Patient* - *Focused* *Care* ”[ *Title* / *Abstract* ])) *OR* (“ *Patient Focused Care* ”[ *Title* / *Abstract* ])) *OR* (“ *Patient* - *Centered* *Nursing* ”[ *Title* / *Abstract* ])) *OR* (“ *Patient Centered Nursing* ”[ *Title* / *Abstract* ]))) *OR* (((((((((((((“ *Continuity of Patient Care* ”[MeSH *Terms* ]) *OR* (“ *Care Continuity* , *Patient* ”[MeSH *Terms* ])) *OR* (“ *Patient Care Continuity* ”[MeSH *Terms* ])) *OR* (“ *Continuum of Care* ”[MeSH *Terms* ])) *OR* (“ *Care Continuum* ”[MeSH *Terms* ])) *OR* (“ *Continuity of Care* ”[MeSH *Terms* ])) *OR* (“ *Care Continuity* ”[MeSH *Terms* ])) *OR* (“ *Continuity of Patient Care* ”[ *Title* / *Abstract* ])) *OR* (“ *Patient Care Continuity* ”[ *Title* / *Abstract* ])) *OR* (“ *Continuum of Care* ”[ *Title* / *Abstract* ])) *OR* (“ *Care Continuum* ”[ *Title* / *Abstract* ])) *OR* (“ *Continuity of Care* ”[ *Title* / *Abstract* ])) *OR* (“ *Care Continuity* ”[ *Title* / *Abstract* ]))) *OR* ((((((((((“ *Transitional Care* ”[MeSH *Terms* ]) *OR* (“ *Care* , *Transitional* ”[MeSH *Terms* ])) *OR* (“ *Cares* , *Transitional* ”[MeSH *Terms* ])) *OR* (“ *Transitional Cares* ”[MeSH *Terms* ])) *OR* (“ *Transition Care* ”[MeSH *Terms* ])) *OR* (“ *Transition Cares* ”[MeSH *Terms* ])) *OR* (“ *Transitional Care* ”[ *Title* / *Abstract* ])) *OR* (“ *Transitional Cares* ”[ *Title* / *Abstract* ])) *OR* (“ *Transition Care* ”[ *Title* / *Abstract* ])) *OR* (“ *Transition Cares* ”[ *Title* / *Abstract* ]))) *OR* (((((((((((((((((((((((((((((((((((((“ *Patient Transfer* ”[MeSH *Terms* ]) *OR* (“ *Patient Transfers* ”[MeSH *Terms* ])) *OR* (“ *Transfer* , *Patient* ”[MeSH *Terms* ])) *OR* (“ *Transfers* , *Patient* ”[MeSH *Terms* ])) *OR* (“ *Patient Transition* ”[MeSH *Terms* ])) *OR* (“ *Patient Transitions* ”[MeSH *Terms* ])) *OR* (“ *Transition* , *Patient* ”[MeSH *Terms* ])) *OR* (“ *Transitions* , *Patient* ”[MeSH *Terms* ])) *OR* (“ *Care Transition* ”[MeSH *Terms* ])) *OR* (“ *Care Transitions* ”[MeSH *Terms* ])) *OR* (“ *Transition* , *Care* ”[MeSH *Terms* ])) *OR* (“ *Transitions* , *Care* ”[MeSH *Terms* ])) *OR* (“ *Transition of Care* ”[MeSH *Terms* ])) *OR* (“ *Health Care Transition* ”[MeSH *Terms* ])) *OR* (“ *Care Transition* , *Health* ”[MeSH *Terms* ])) *OR* (“ *Care Transitions* , *Health* ”[MeSH *Terms* ])) *OR* (“ *Health Care Transitions* ”[MeSH *Terms* ])) *OR* (“ *Transition* , *Health Care* ”[MeSH *Terms* ])) *OR* (“ *Transitions* , *Health Care* ”[MeSH *Terms* ])) *OR* (“ *Patient Turfing* ”[MeSH *Terms* ])) *OR* (“ *Patient Turfings* ”[MeSH *Terms* ])) *OR* (“ *Turfing* , *Patient* ”[MeSH *Terms* ])) *OR* (“ *Turfings* , *Patient* ”[MeSH *Terms* ])) *OR* (“ *Patient Dumping* ”[MeSH *Terms* ])) *OR* (“ *Dumping* , *Patient* ”[MeSH *Terms* ])) *OR* (“ *Patient Transfer* ”[ *Title* / *Abstract* ])) *OR* (“ *Patient Transfers* ”[ *Title* / *Abstract* ])) *OR* (“ *Patient Transition* ”[ *Title* / *Abstract* ])) *OR* (“ *Patient Transitions* ”[ *Title* / *Abstract* ])) *OR* (“ *Care Transition* ”[ *Title* / *Abstract* ])) *OR* (“ *Care Transitions* ”[ *Title* / *Abstract* ])) *OR* (“ *Transition of Care* ”[ *Title* / *Abstract* ])) *OR* (“ *Health Care Transition* ”[ *Title* / *Abstract* ])) *OR* (“ *Health Care Transitions* ”[ *Title* / *Abstract* ])) *OR* (“ *Patient Turfing* ”[ *Title* / *Abstract* ])) *OR* (“ *Patient Turfings* ”[ *Title* / *Abstract* ])) *OR* (“ *Patient Dumping* ”[ *Title* / *Abstract* ]))) *OR* (“ *Transition in care* ”)) *AND* (((((((“ *Critical Care* ”[MeSH *Terms* ]) *OR* (“ *Care* , *Critical* ”[MeSH *Terms* ])) *OR* (“ *Intensive Care* ”[MeSH *Terms* ])) *OR* (“ *Care* , *Intensive* ”[MeSH *Terms* ])) *OR* (“ *Critical Care* ”[ *Title* / *Abstract* ])) *OR* (“ *Intensive Care* ”[ *Title* / *Abstract* ]) *OR* (((((((“ *Intensive Care Units* ”[MeSH *Terms* ]) *OR* (“ *Intensive Care Unit* ”[MeSH *Terms* ])) *OR* (“ *Unit* , *Intensive Care* ”[MeSH *Terms* ])) *OR* (“ *ICU Intensive Care Units* ”[MeSH *Terms* ])) *OR* (“ *Intensive Care Units* ”[ *Title* / *Abstract* ])) *OR* (“ *Intensive Care Unit* ”[ *Title* / *Abstract* ])) *OR* (“ *ICU Intensive Care Units* ”[ *Title* / *Abstract* ])))) *AND* (( *Perception* [ *Title* / *Abstract* ]) *OR* *(Experience* [ *Title* / *Abstract* ]))

### Seleção das fontes de evidência

Os achados foram carregados no gerenciador de referências EndNote *Web* para remoção automática das duplicatas. Em seguida, utilizou-se o *software* Rayyan para dar continuidade ao processo, ainda com a exclusão manual dos trabalhos duplicados.

Os títulos e os resumos foram avaliados por duas revisoras de forma cega e independente para seleção em relação aos critérios de elegibilidade especificados. Quaisquer desacordos entre as revisoras no decorrer da seleção foram resolvidos por consenso. Quando não foi possível um acordo, uma terceira pesquisadora foi consultada para a decisão final.

### Extração dos dados

O texto completo dos registros selecionados foi avaliado detalhadamente por duas pesquisadoras independentes. Uma ficha de extração de dados foi elaborada pelas autoras, contendo os seguintes itens: autor(es), título, periódico, ano, volume, número, página(s), *digital object identifier* ou *link* de acesso, país onde o trabalho foi conduzido, tipo de publicação, objetivo(s), critério(s) de inclusão e de exclusão, amostra, coleta e análise dos dados, resultados principais, etapas para a transição do cuidado entre terapia intensiva e unidade de internação e evidência(s) identificada(s) a partir da lista de referências.

Duas pesquisadoras familiarizaram-se com a ficha em um teste-piloto, isto é, ao menos duas fontes foram consultadas para garantia da adequação do formulário ao propósito desta investigação.

Os dados foram extraídos por uma revisora e uma segunda pesquisadora verificou as informações colhidas. Quaisquer desacordos entre as pesquisadoras foram avaliados por uma terceira revisora.

### Tratamento e análise dos dados

A síntese dos dados extraídos foi organizada sob a forma de figuras e análise descritiva, visando-se um alinhamento com o objetivo e com a questão de pesquisa. Ainda, uma análise de conteúdo qualitativa básica, empregando-se abordagem dedutiva^(20)^, foi desenvolvida para categorizar as experiências dos pacientes e seus familiares conforme as etapas para a transição do cuidado entre terapia intensiva e unidade de internação, a saber: avaliação, planejamento, execução e acompanhamento^(8)^.

### Aspectos éticos

Como se trata de uma revisão baseada em dados secundários, de domínio público e disponíveis na literatura, a apreciação ética é prescindível. Porém, ressalta-se que os direitos autorais foram respeitados com as devidas citações e referências dos estudos consultados.

## Resultados

A busca nas bases de dados identificou 7.646 registros e outros sete foram considerados potencialmente elegíveis por meio das listas de referências das fontes selecionadas, permanecendo 30 estudos na amostra final, conforme elucidado na Figura 2.

**Figura 2-:** Fluxograma PRISMA^(21)^. Porto Alegre, RS, Brasil, 2024

^*^PubMed^®^ (n = 1.328); Biblioteca Virtual em Saúde (n = 878); *Cumulative Index to Nursing and Allied Health Literature* (n = 1.374); *Cochrane Database of Systematic Reviews* (n = 138); Embase (n = 1.555); Scopus (n = 1.817); *Web of Science* (n = 505); Biblioteca Digital Brasileira de Teses e Dissertações (n = 13); *Networked Digital Library of Theses and Dissertations* (n = 38)

As características dos registros incluídos, segundo ano, país, tipo de estudo, objetivo(s) e amostra, encontram-se sumarizadas na Figura 3.

**Figura 3-:** Caracterização dos estudos incluídos. Porto Alegre, RS, Brasil, 2024

**Autor(es), ano**	**País**	**Tipo de estudo**	**Objetivo(s)**	**Amostra**
Leith, 1999 ^( 22 )^	Canadá	Qualitativo	Descrever as percepções do paciente e seus familiares sobre a transferência de uma UTI*.	Pacientes (n = 53) e familiares (n = 35)
Odell, 2000 ^( 23 )^	Inglaterra	Qualitativo	Analisar como pacientes se sentem ao serem transferidos da UTI* para a UI ^†^ após a recuperação de uma doença ou lesão crítica.	Pacientes (n = 6)
McKinney, et al., 2002 ^( 24 )^	Irlanda do Norte	Qualitativo	Explorar a experiência vivida por pacientes na transferência da UTI* para a UI ^†^ .	Pacientes (n = 6)
Chaboyer, et al., 2005 ^( 25 )^	Austrália	Qualitativo	Explorar as percepções individuais e coletivas de um grupo de pacientes e seus familiares sobre a transferência da UTI*.	Pacientes (n = 7) e familiares (n = 6)
Strahan, et al., 2005 ^( 26 )^	Irlanda do Norte	Qualitativo	Explorar e descrever as experiências vividas pelos pacientes após a sua transferência da UTI*.	Pacientes (n = 10)
Field, et al., 2008 ^( 27 )^	Inglaterra	Qualitativo	Explorar e examinar os relatos de ex-pacientes de UTI* para identificar causas adicionais de estresse de realocação.	Pacientes (n = 34)
Bench, et al., 2010 ^( 28 )^	Inglaterra	Metassíntese	Identificar os fatores mais significativos que impactam a experiência do paciente no progresso e na recuperação de uma doença crítica durante o primeiro mês após a alta da UTI ^*^ , e discutir esses fatores em relação ao desenvolvimento de estratégias eficazes de apoio à alta da UTI*.	Registros (n = 10) contemplando pacientes e familiares
Bench, et al., 2011 ^( 29 )^	Inglaterra	Qualitativo	Buscar a opinião de pacientes, familiares e profissionais de saúde sobre os métodos mais eficazes de fornecer informações sobre a alta da UTI*, o conteúdo das informações necessárias, os benefícios e as limitações das informações existentes e as potenciais implicações em termos de recursos.	Pacientes (n = 11), familiares (n = 8) e profissionais de saúde (n = 23)
Forsberg. et al., 2011 ^( 30 )^	Suécia	Qualitativo	Descrever as experiências das pessoas que receberam cuidados na UTI* e foram transferidas para uma UI ^†^ .	Pacientes (n = 10)
Calatayud, et al., 2013 ^( 31 )^	Espanha	Revisão bibliográfica	Identificar, explorar e apresentar criticamente as evidências existentes sobre como os pacientes, familiares e profissionais de enfermagem experimentam o processo de transição da UTI* para a UI ^†^ , e analisar as possíveis intervenções existentes para o desenvolvimento de um processo de transição ótimo.	Registros (n = 23) contemplando pacientes e familiares
Cullinane, et al., 2013 ^( 32 )^	Inglaterra	Revisão da literatura	Analisar as evidências sobre a transferência de pacientes da UTI* para a UI ^†^ .	Registros (n = 8) contemplando pacientes e familiares
Cypress, 2013 ^( 33 )^	Estados Unidos	Revisão da literatura	Revisar sistematicamente os efeitos da transferência ou alta da UTI* para unidades de internação médico-cirúrgicas em pacientes adultos gravemente enfermos, seus familiares e enfermeiros.	Registros (n = 27) contemplando pacientes, familiares e profissionais de saúde
Häggström, et al., 2014 ^( 34 )^	Suécia	Método misto	Investigar a percepção dos familiares sobre a qualidade do cuidado durante o processo de transferência de um paciente de uma UTI* para uma UI ^†^ .	Familiares (n = 65)
Ramsay, et al., 2014 ^( 35 )^	Escócia	Método misto	Explorar as necessidades psicossociais dos pacientes que recebem alta da UTI*, à medida em que estas são captadas utilizando a teoria das transições e o potencial desenvolvimento do papel dos serviços de proximidade, acompanhamento e ligação dos cuidados intensivos.	Pacientes (n = 20)
Gill, et al., 2016 ^( 36 )^	Canadá	Qualitativo	Explorar as experiências e perspectivas dos doentes e das suas famílias sobre os cuidados na UTI* sem a influência indevida dos prestadores de cuidados ou dos investigadores “tradicionais”.	Pacientes e familiares (n = 32)
Stelfox, et al., 2017 ^( 37 )^	Canadá	Qualitativo	Compreender os atuais procedimentos de transferência e as experiências dos pacientes e dos profissionais (médicos e enfermeiros da UTI* e da UI ^†^ ), em uma visão de 360 graus, sobre a transferência da UTI* para a UI ^†^ , a fim de informar as recomendações para melhorar os cuidados.	Pacientes (n = 451)
Antonio, et al., 2018 ^( 38 )^	Brasil	Qualitativo	Compreender, na perspectiva dos acompanhantes, a transição dos pacientes que receberam alta das UTIs*.	Familiares (n = 30)
de Grood, et al., 2018 ^( 39 )^	Canadá	Qualitativo	Avaliar as perspectivas dos pacientes e dos prestadores de cuidados sobre barreiras e facilitadores para transferências de alta qualidade e recomendações para melhorar o processo de transferência.	Pacientes, familiares e profissionais de saúde (n = 35)
Koilor, et al., 2018 ^( 40 )^	Estados Unidos	Qualitativo	Caracterizar a experiência familiar inicial de recuperação de doença crítica traumática.	Familiares (n = 13)
King, et al., 2019 ^( 41 )^	Canadá	Revisão de escopo	Descrever que tipos de suporte os pacientes precisam após a alta da UTI* e de que forma as necessidades de suporte diferem ao longo do *continuum* de recuperação (da alta da UTI* até a recuperação de longo prazo baseada na comunidade).	Registros (n = 32) contemplando pacientes
Herling, et al., 2020 ^( 42 )^	Dinamarca	Qualitativo	Explorar as experiências de pacientes da UTI* e seus familiares durante a transição para a UI ^†^ , a fim de encontrar maneiras de apoiá-los durante o processo de transição.	Pacientes (n = 10) e familiares (n = 4)
Op ’t Hoog, et al., 2020 ^( 43 )^	Holanda	Qualitativo	Obter uma compreensão mais profunda das experiências e necessidades dos familiares de pacientes de UTI* durante a transição da UTI* para uma UI ^†^ .	Familiares (n = 13)
Ghorbanzadeh, et al., 2021 ^( 44 )^	Irã	Qualitativo	Investigar a compreensão e os desafios que os pacientes enfrentam durante seu processo de transição da UTI*.	Pacientes e familiares (n = 8) e profissionais de saúde (n = 14)
Lee, et al., 2021 ^( 45 )^	Coreia do Sul	Qualitativo	Obter uma compreensão aprofundada da experiência de transferência de pacientes de UTI* na Coreia do Sul por meio de uma análise fenomenológica.	Pacientes (n = 15)
Cuzco, et al., 2022 ^( 46 )^	Espanha	Qualitativo	Descrever a experiência dos pacientes durante a transição da UTI* para a UI ^†^ .	Pacientes (n = 48)
Zhan, et al., 2022 ^( 47 )^	China	Qualitativo	Explorar as experiências e necessidades dos cuidadores familiares em relação aos cuidados de transição durante a transferência de uma UTI* para uma UI ^†^ .	Familiares (n = 15)
Cuzco, et al., 2023 ^( 48 )^	Espanha	Qualitativo	Explorar as características da transição da UTI* para a UI ^†^ a partir da experiência vivida pelo paciente, e identificar a terapêutica de enfermagem durante a transferência dos pacientes da UTI* para a UI ^†^ .	Pacientes (n = 48)
Gullberg, et al., 2023 ^( 49 )^	Suécia	Qualitativo	Descrever as experiências dos pacientes na preparação para a transferência de uma UTI* para uma UI ^†^ .	Pacientes (n = 14)
Pastor, et al., 2023 ^( 50 )^	Espanha	Qualitativo	Conhecer os sentimentos e emoções que emergem da experiência de alta de uma UTI*, e identificar os fatores que desencadeiam bem-estar e desconforto emocional nos pacientes.	Pacientes (n = 20)
Meiring- Noordstra, et al., 2024 ^( 51 )^	Holanda	Qualitativo	Explorar as experiências de familiares de pacientes internados em UTI* com quadro agudo na transição da UTI* para uma UI ^†^ e depois para casa.	Familiares (n = 12)

*UTI = Unidade de Terapia Intensiva; ^†^UI = Unidade de Internação.

Identificaram-se publicações de 1999 a 2024, com destaque para os anos de 2023 (n = 4), 2018 (n = 3) e 2013 (n = 3). Em relação à procedência, evidenciaram-se estudos conduzidos por pesquisadores do Reino Unido (n = 7), do Canadá (n = 5) e da Espanha (n = 4). Quanto ao tipo de estudo, os autores desenvolveram majoritariamente pesquisas primárias qualitativas (n = 21) para abordar a temática, contemplando como população prevalente de interesse os pacientes (n = 13), seguida da díade (pacientes e familiares) (n = 7) e somente familiares (n = 6). Os estudos incluídos foram conduzidos predominantemente por autores com formação em enfermagem e medicina, abrangendo profissionais assistenciais, gestores, docentes e pesquisadores.

Os procedimentos de coleta de dados dos estudos incluídos foram realizados em momentos distintos: na unidade de internação após a alta da UTI, podendo variar de um a sete dias posteriores à transferência^(23-24,26,37,40,42-43,45-48)^; e no domicílio, por contato telefônico, videochamada ou no retorno à instituição conforme combinação, de meses^(25,28,30,35,40,42,49,51)^ a anos^(27,29,36)^ após a alta hospitalar.

As experiências dos pacientes e seus familiares, retratadas nas publicações mapeadas, categorizadas conforme as etapas para a transição do cuidado entre terapia intensiva e unidade de internação, estão representadas na Figura 4. Uma visão geral completa das experiências está disponível no repositório de dados SciELO Data (https://doi.org/10.48331/scielodata.PDUSZX).

**Figura 4-:** Experiências de pacientes e familiares na transição do cuidado entre terapia intensiva e unidade de internação. Porto Alegre, RS, Brasil, 2025

*UI = Unidade de Internação; ^†^UTI = Unidade de Terapia Intensiva

## Discussão

As experiências dos pacientes e seus familiares foram categorizadas, de forma inovadora, à luz das etapas descritas para a transição do cuidado entre terapia intensiva e unidade de internação: avaliação para a alta, planejamento da alta, execução da alta e acompanhamento após a alta^(8)^. Essa estrutura analítica permitiu identificar pontos críticos e recorrentes do processo, destacando a transição como uma fase sensível da hospitalização, marcada por elevada carga emocional e por lacunas informacionais transversais a todas as etapas. A ausência de preparo adequado para a alta, associada a deficiências na comunicação entre as unidades, revelam-se como fatores que dificultam a continuidade assistencial e a adaptação dos indivíduos ao novo ambiente.

Os achados desta revisão contribuem para a sistematização de evidências que conferem voz aos próprios indivíduos que vivenciam a transição, revelando um cenário persistente de fragilidades ainda pouco superadas: ao longo de mais de duas décadas, predominaram experiências negativas de pacientes e familiares nesse contexto. Além disso, os achados sinalizam direções promissoras para investigações futuras, voltadas ao aprofundamento da compreensão das demandas específicas dos indivíduos, bem como à avaliação do impacto das suas experiências na recuperação e na continuidade do cuidado.

Pacientes e familiares experienciam uma transição situacional e organizacional. Situacional, pois vivenciam uma transição de saúde-doença desde a admissão, diagnóstico crítico, recuperação e alta da terapia intensiva, e organizacional, ao transitarem entre duas unidades com configurações distintas, inerentes aos seus respectivos níveis assistenciais. Essa transição, por si só, expõe o indivíduo a uma situação de vulnerabilidade^(1,52)^.

Na etapa de *avaliação para a alta*, observa-se um movimento crescente entre os profissionais em direção ao estabelecimento de critérios objetivos para essa decisão, fundamentados na estabilidade orgânica do indivíduo e na capacidade da unidade receptora. No entanto, a visão do paciente e da família é pouco debatida^(12,53-54)^, corroborando as manifestações de falta de envolvimento e distanciamento sobre a definição tomada pelo profissional. Diferentemente do contexto de alta hospitalar, que já conta com um instrumento validado e traduzido para diversos idiomas para avaliar o preparo na percepção do paciente^(55-56)^.

Pacientes críticos geralmente perdem autonomia e se encontram em uma situação de dependência para atividades básicas de vida diária. O conhecimento e a preparação para a mudança facilitam o empoderamento do indivíduo para se adaptar ao novo contexto e retomar sua independência. A educação em saúde está entre as principais intervenções de enfermagem nesse processo de transição^(57-58)^. Trabalhos envolvendo o desenvolvimento de um modelo^(59)^ ou um pacote de medidas^(60)^ para a transição do cuidado na alta da UTI consideram a informação como facilitadora desse processo, uma vez que capacita, fornece ferramentas para uma participação ativa e promove relacionamentos colaborativos.

O compartilhamento de informações graduais, repetidas e apoiadas por material escrito e espaços de escuta para o esclarecimento de dúvidas, conduzidos de forma respeitosa, são alicerces de transições bem sucedidas^(8,61-62)^. Iniciativas para envolver e capacitar os indivíduos são relatadas para a alta da UTI^(63-64)^, para a educação centrada na família^(65)^ e para os cuidados após esse momento crítico^(66)^. Todavia, em consonância com os resultados encontrados nesta revisão, outras pesquisas apontam que pacientes e familiares são frequentemente excluídos do *planejamento da alta*, recebendo informações insuficientes e divergentes, com termos técnicos e pouco tempo dedicado a essa atividade^(6,8)^.

O intervalo de tempo ideal para esse planejamento ainda é desconhecido, mas, períodos inferiores a seis horas e superiores a 48 horas têm sido considerados por profissionais e gestores de terapia intensiva^(12)^. Entretanto, a pressão pela disponibilidade de leitos^(67-68)^, aliada à elevada carga de trabalho^(69)^, com frequência criam um ambiente conturbado de tal forma que as oportunidades instrutivas, muitas vezes, são menosprezadas^(6,69)^.

Barreiras de comunicação e preparação inadequada são fatores relacionados ao diagnóstico de enfermagem “síndrome de estresse de realocação”, definido como uma perturbação fisiológica e/ou psicossocial associada à transferência de um ambiente para outro, caracterizado, entre outros, por preocupação, insegurança, ansiedade e medo^(70)^ – sentimentos consistentemente mencionados na amostra selecionada. Pesquisadores validaram uma ferramenta de autorrelato para avaliar o estresse de realocação em pacientes após a transferência da UTI, com o propósito de identificar problemas psicológicos durante a mudança e auxiliar os profissionais na elaboração de cuidados centrados na pessoa, visando minimizar as perturbações associadas ao período de transição^(71)^.

A transferência de cuidados à beira leito para a *execução da alta*^(69,72)^ foi identificada como experiência positiva por pacientes e familiares e vem sendo relatada como estratégia favorável à transição do cuidado. Em contrapartida, atrasos devido à indisponibilidade de leitos na unidade de internação^(73-75)^, transferências em finais de semana^(75-77)^, nas trocas de turno^(77)^ ou à noite^(77-78)^ contribuíram para a insatisfação dos indivíduos e têm sido associadas a desfechos negativos.

Quanto à comunicação entre as equipes da UTI e da unidade de internação, tanto os profissionais^(79)^ quanto os pacientes e familiares apontaram fragilidades nesse processo. Estudos mostram que, a despeito das orientações, a comunicação carece de padronização e, comumente, os dados transmitidos ficam a critério dos profissionais da UTI^(8,80-82)^. A literatura também ressalta que as práticas atuais estão aquém do ideal, com discrepâncias entre as informações transmitidas pela equipe intensivista e as necessidades daqueles que as recebem na unidade de internação^(8,69,77,80,82)^.

Os profissionais da UTI frequentemente superestimam a capacidade da equipe receptora^(13-14,79,82)^, que, por sua vez, percebe essa transição como uma grande responsabilidade. Essa percepção é intensificada pelo recebimento de pacientes instáveis, com históricos complexos, tratamentos pouco claros, transferidos precocemente e com preparação insuficiente para deixarem o ambiente seguro da terapia intensiva. O desafio torna-se ainda maior quando a equipe receptora se sente inexperiente ou carece de qualificação para atender esse perfil de paciente^(67,69)^, uma fragilidade percebida tanto pelos pacientes quanto pelos familiares. Enfermeiros de unidades de internação sugeriram como estratégias de melhoria o contato prévio com enfermeiros intensivistas para troca de informações e a possibilidade de vivenciar um período na UTI para observar os procedimentos realizados e aprofundar a compreensão sobre o cuidado crítico^(67)^. Essa prática também beneficia pacientes e familiares ao oportunizar um primeiro contato com os profissionais da equipe receptora, antes da transferência, favorecendo a continuidade e a humanização do cuidado^(83)^.

Entre as publicações analisadas, a redução abrupta e mal comunicada do cuidado assistencial sobressaiu-se como uma experiência estressante. Quando não há orientação sobre as diferenças esperadas na intensidade de suporte entre as unidades, a percepção do indivíduo pode ser distorcida, levando a comparações irreais. Além disso, há um descompasso entre a dependência dos cuidados intensivos recebidos na UTI e a necessidade de autossuficiência para executar as mesmas tarefas na unidade de internação, sem tempo suficiente para essa adaptação. Familiares frequentemente relatam como adversidades relacionadas à alta da terapia intensiva a perda de informações, a redução no monitoramento e a sensação de transferência precoce^(11)^. Para melhorar a qualidade da transição e garantir a segurança do paciente, é fundamental implementar práticas como a redução gradual do nível de cuidado ainda na UTI, incluindo a remoção de dispositivos invasivos desnecessários, a retirada progressiva da monitorização multiparamétrica e a avaliação da mobilidade^(6,84-85)^.

Ademais, a presença e o envolvimento da família são imprescindíveis ao processo de alta da UTI. Muitos indivíduos vivenciam a alta da terapia intensiva com nível e/ou conteúdo de consciência alterado, com déficit cognitivo e/ou de comunicação importante ou dependentes para atividades básicas de vida diária, e os familiares atuam como um elo de suporte emocional e informacional, auxiliando sua adequação à nova fase de recuperação, e, variadas vezes, assumindo papéis decisórios com relação a condutas e tratamentos^(6,8,86)^. Eles buscam ativamente ser reconhecidos como parte essencial no cuidado de seu familiar^(87)^.

Programas de *acompanhamento após a alta* da UTI têm sido implementados de diversas formas. Equipes de transição do cuidado monitoram o paciente na unidade de internação por pelo menos 24 horas após a alta da terapia intensiva^(88-90)^, enquanto serviços de enfermagem de ligação realizam atividades voltadas para a segurança, manejo de vias aéreas, prevenção de infecções e quedas, cuidados com a pele e educação e suporte aos pacientes e familiares^(91-92)^. Embora revisões sistemáticas e metanálises indiquem evidências limitadas sobre a efetividade dessas intervenções na redução de readmissões e mortalidade^(93-94)^, os familiares relataram que suas necessidades foram atendidas e consideram valiosa a atuação dos enfermeiros de ligação, especialmente pelo suporte oferecido durante a transição^(95)^.

Um estudo destacou os benefícios de um Grupo de Cuidados Pós-Intensivos, no qual enfermeiros da UTI ofereceram cuidados adicionais aos pacientes mais vulneráveis na unidade de internação. Esses cuidados foram direcionados às necessidades físicas e psicológicas dos pacientes, incluindo visitas mais frequentes, quando necessário, o que promoveu maior confiança e redução da ansiedade tanto dos pacientes quanto de seus familiares. As principais atividades do grupo concentraram-se na prevenção, com a detecção precoce de sinais de deterioração, a mitigação de eventos adversos e a promoção da troca de conhecimento entre as equipes, com foco no melhor interesse do paciente^(96)^.

Adicionalmente, os 30 estudos analisados foram publicados de maneira equilibrada ao longo do tempo e conduzidos por diferentes perfis profissionais, atuantes na assistência, gestão, docência e pesquisa. Essa distribuição evidencia uma demanda constante por produção de conhecimento sobre o tema, bem como o interesse compartilhado entre distintos segmentos da prática e da ciência em compreender e qualificar esse processo. Embora a predominância recaia sobre autores da enfermagem e da medicina, a complexidade do fenômeno investigado exige abordagens integradas e o desenvolvimento de estudos futuros que incorporem a perspectiva da equipe multidisciplinar.

Mais da metade dos estudos foram desenvolvidos no continente europeu, refletindo uma concentração na produção científica em poucos países e ressaltando a necessidade de explorar essa temática em outras realidades. Revisões de escopo que abordaram a transição do cuidado entre UTI e unidade de internação, de pacientes adultos^(6)^ e de neonatais, pediátricos e adultos^(8)^, também apontaram a Europa e a América do Norte como as principais origens da maioria das publicações incluídas.

Outro tópico relevante sobre as características dos estudos é a variabilidade nos períodos de coleta de dados adotados pelos pesquisadores. Não há descrito o intervalo de tempo ideal, ao que parece, autores consideram períodos-chave para capturar impressões de interesse contextuais. Por exemplo, para compreender como os pacientes e suas famílias vivenciam o ambiente de terapia intensiva e seu impacto na recuperação, dados foram coletados na primeira semana após a alta da UTI^(97)^, já para investigar os efeitos da realidade virtual na saúde mental, na qualidade percebida e na satisfação após tratamento intensivo entre sobreviventes da COVID-19, as informações foram coletadas até seis meses após a alta hospitalar^(98)^.

Entre as limitações desta revisão de escopo estão a restrição de idioma e a inacessibilidade ao texto completo de quatro registros, o que pode ter resultado em uma amostra incompleta e menos representativa. Igualmente, embora a extração de dados tenha sido realizada de forma sistemática por duas revisoras, é possível que não tenha sido totalmente precisa e exaustiva.

Esta revisão contribui para o avanço do conhecimento científico para a área de saúde e de enfermagem ao reunir evidências provenientes de diferentes contextos e temporalidades e ampliar a visibilidade das etapas da transição do cuidado entre terapia intensiva e unidade de internação sob a perspectiva de pacientes e familiares. A representação criada (Figura 4) pode subsidiar atividades de educação permanente em saúde, ao sintetizar e operacionalizar o conhecimento disponível, com ênfase nas fragilidades identificadas em cada etapa do processo. Os achados implicam na necessidade de aprimorar as estratégias atualmente adotadas, mediante intervenções estruturadas e oportunas, com vistas a qualificar a continuidade assistencial e reduzir as experiências negativas ainda recorrentes ao longo dessa trajetória.

## Conclusão

A transição do cuidado entre terapia intensiva e unidade de internação é um processo complexo e multifacetado, frequentemente associado a desafios que afetam pacientes, familiares e equipes assistenciais. As experiências descritas nas publicações mapeadas destacam esse momento como um evento marcante da internação hospitalar, frequentemente acompanhado de impacto emocional. A falta de informação foi identificada em todas as etapas do processo, com relatos recorrentes de despreparo e percepções de abandono, especialmente em caso de transferência repentina. Além disso, a comunicação inadequada entre as partes envolvidas resultou em descompassos assistenciais, dificultando a adaptação dos indivíduos e comprometendo a transição.

Nesse contexto, a educação em saúde se destaca como uma das principais intervenções de enfermagem para reduzir as lacunas identificadas. Oferecer informações claras e oportunas, explicar as diferenças esperadas entre os níveis assistenciais e adotar uma comunicação estruturada são estratégias primordiais. Ratifica-se, como contribuição relevante desta revisão, a categorização das experiências de pacientes e familiares entre as etapas da transição do cuidado entre terapia intensiva e unidade de internação, a qual proporciona uma visualização clara e acessível dos principais pontos críticos do processo. Essa sistematização poderá ser empregada como um recurso estratégico em atividades de educação permanente em saúde, ao fomentar a tradução do conhecimento em prática e indicar aspectos que demandam atenção em cada etapa.

Ademais, é essencial promover uma cultura organizacional com responsabilidades claramente definidas entre as categorias profissionais envolvidas no preparo do paciente e da família. Essa abordagem deve substituir práticas isoladas e minimizar a dependência de ações subjetivas, fortalecendo um ambiente de cuidado colaborativo.

Avançar na compreensão desse tema exige o desenvolvimento de estratégias baseadas em evidências, incluindo modelos integrados de transição com comunicação padronizada, programas educativos para preparar pacientes e familiares antes da transferência e instrumentos validados para mensurar percepções sobre preparo, satisfação e segurança durante o processo. Pesquisas futuras podem contribuir para identificar práticas que promovam autonomia, empoderamento e segurança e compreender melhor o impacto da transição do cuidado nas experiências vividas. Dessa forma, será possível não apenas reduzir o estresse e a vulnerabilidade associados a esse momento, mas também garantir uma assistência equitativa, eficiente e centrada na pessoa.

## Data Availability

O conjunto de dados deste artigo está disponível na página da RLAE no repositório SciELO Data, no link https://doi.org/10.48331/scielodata.PDUSZX

# Translation: Experiencias de transición de pacientes y familiares al alta de la terapia intensiva: revisión de alcance*

**DOI:** 10.1590/1518-8345.7919.4761

**Authors:** Paula Buchs Zucatti, Clediane Rita Portalupi da Trindade, Rosana Evangelista-Poderoso, Edinêis de Brito Guirardello, Maria Alice Dias da Silva Lima

**Affiliations:** 1 Universidade Federal do Rio Grande do Sul, Escola de Enfermagem e de Saúde Coletiva, Porto Alegre, RS, Brasil. Universidade Federal do Rio Grande do Sul Escola de Enfermagem e de Saúde Coletiva RS Porto Alegre Brasil; 2 Hospital de Clínicas de Porto Alegre, Centro de Tratamento Intensivo, Porto Alegre, RS, Brasil. Hospital de Clínicas de Porto Alegre Centro de Tratamento Intensivo RS Porto Alegre Brasil; 3 Universidade Estadual de Campinas, Biblioteca da Faculdade de Ciências Médicas, Campinas, SP, Brasil. Universidade Estadual de Campinas Biblioteca da Faculdade de Ciências Médicas SP Campinas Brasil; 4 Universidade Estadual de Campinas, Faculdade de Enfermagem, Campinas, SP, Brasil. Universidade Estadual de Campinas Faculdade de Enfermagem SP Campinas Brasil; 5 Becaria del Conselho Nacional de Desenvolvimento Científico e Tecnológico (CNPq), Brasil. Becaria del Conselho Nacional de Desenvolvimento Científico e Tecnológico Brasil

**Keywords:** Alta del Paciente, Atención Dirigida al Paciente, Continuidad de la Atención al Paciente, Cuidado de Transición, Participación del Paciente, Transferencia de Pacientes

## Abstract

mapear las evidencias disponibles sobre las experiencias de pacientes adultos y familiares en relación con la transición del cuidado entre terapia intensiva y unidad de hospitalización.

revisión de alcance realizada según las directrices del *Joanna BriggsInstitute*. Se incluyeron estudios primarios, revisiones, disertaciones y tesis, publicados en portugués, inglés o español, sin límite temporal. La búsqueda final se realizó en portales y bases de datos en salud y bibliotecas digitales. La selección fue realizada por dos revisoras de forma ciega e independiente. Los datos fueron extraídos mediante un guion y presentados en figuras, análisis descriptivo y análisis cualitativo deductivo, categorizando las vivencias entre las etapas de evaluación, planificación, ejecución y acompañamiento posterior al alta.

treinta artículos compusieron la muestra. Las experiencias mapeadas evidencian la transición como un momento crítico de la hospitalización, marcado por impacto emocional y fragilidades informacionales. Relatos de falta de preparación, sensación de abandono y fallas de comunicación comprometen la continuidad del cuidado y la adaptación de pacientes y familiares.

las experiencias descritas señalan la transición del cuidado entre terapia intensiva y unidad de hospitalización como un proceso complejo y multifacético. Las brechas identificadas demandan estrategias de comunicación estructurada, información oportuna y alineamiento entre las unidades.

## Destacados

**(1)** La transición entre la terapia intensiva y la unidad de hospitalización es compleja y desafiante. **(2)** Pacientes y familiares experimentan reducción abrupta y discontinuidad del cuidado. **(3)** La comunicación imprecisa o insuficiente está presente en todas las etapas del proceso. **(4)** Presencia e involucramiento de la familia como vínculo de apoyo emocional e informacional.

## Introducción

El alta de la Unidad de Cuidados Intensivos (UCI) hacia la unidad de hospitalización representa un cambio significativo, marcando un proceso de transición importante para el paciente y su familia. Mientras el cambio es un evento específico y delimitado en el tiempo, la transición es un proceso continuo, caracterizado por una serie de respuestas. Durante este período, los individuos enfrentan contextos, sensaciones y emociones desconocidas, además de lidiar con incertidumbres sobre el futuro, lo que los coloca en una condición de vulnerabilidad^(1)^.

La forma en que la persona interpreta el cambio, su significado y la disponibilidad de apoyo y de recursos repercuten directamente en sus experiencias, pudiendo favorecer o dificultar su recuperación^(1)^. La experiencia del paciente resulta de la suma de todas las interacciones moldeadas por la cultura organizacional, que influyen en sus percepciones a lo largo del continuo de cuidados^(2)^.

La preparación de los pacientes y sus familiares para afrontar la transición entre los diferentes niveles asistenciales, así como la garantía de la continuidad de los cuidados hasta que los cambios sean incorporados a sus cotidianos desempeñan un papel fundamental. Esta experiencia abarca su comprensión sobre el proceso de alta, su nivel de conocimiento y de involucramiento, las acciones de los profesionales que condicionan las vivencias y la concreción o no de sus expectativas^(1)^.

Además de la fragilidad situacional del individuo, la transferencia de cuidados intensivos a cuidados regulares es reconocida como un punto crítico en el transcurso de la hospitalización. Este proceso es especialmente susceptible a fallas, sea en el desplazamiento del paciente, sea en la transferencia de informaciones entre los sectores del sistema de salud. En este sentido, la transición adecuada del cuidado, comprendida como un conjunto estructurado de acciones realizadas por los profesionales de la salud para asegurar la coordinación y la continuidad de los cuidados, se hace esencial^(3-5)^.

En el escenario de alta de la UCI, la fragmentación del cuidado ha sido asociada a desenlaces negativos, como aumento del estrés y de la insatisfacción de pacientes y familiares, mayor tiempo de hospitalización, eventos adversos, elevación de los costos hospitalarios, reingresos y defunciones^(6-10)^. Además, la experiencia de los individuos en la transición entre terapia intensiva y unidad de hospitalización está influenciada por la planificación, la comunicación y la ejecución de este proceso^(11)^.

Una investigación reciente con profesionales y gestores de terapia intensiva reveló brechas entre el momento en que el paciente deja de necesitar cuidados intensivos y aquel en que puede realizar de forma segura la transición a los cuidados regulares^(12)^. Esta discrepancia refuerza la percepción, relatada por enfermeros de unidades de hospitalización, de que existen “dos mundos distintos”: mientras los profesionales de terapia intensiva tienen expectativas elevadas sobre las capacidades de manejo del equipo de hospitalización, esas demandas pueden representar una amenaza para la seguridad del paciente^(13-14)^. Además, los propios pacientes describen dificultades a lo largo de su recuperación, destacando la falta de informaciones claras y accesibles, así como la ausencia de espacios para la escucha y el esclarecimiento de dudas, lo que compromete la experiencia de cuidado durante este período crítico^(15-17)^.

Un enfoque estructurado implica etapas secuenciales para la transición del cuidado entre terapia intensiva y unidad de hospitalización. Estas etapas incluyen: 1) *evaluación para el alta* con la utilización de puntuaciones específicas para determinar gravedad, riesgos y evolución clínica; 2) *planificación del alta*, que consiste en la síntesis de los problemas de salud, la revisión de las metas de atención, la conciliación de la medicación y la orientación detallada al paciente y a la familia sobre los cuidados recibidos, los planificados, el proceso de alta y el lugar de destino; 3) *ejecución del alta*, que involucra la decisión sobre el momento oportuno para la transferencia, la identificación del equipo receptor y la comunicación efectiva entre las partes implicadas; y 4) *seguimiento tras el alta*, con la prestación de asistencia al paciente en la unidad de hospitalización por un miembro del equipo de la UCI, asegurando la continuidad del cuidado y facilitando la adaptación al nuevo entorno^(8)^.

Aunque las experiencias de los pacientes y sus familiares en el contexto del alta de la UCI han sido ampliamente discutidas en la literatura a lo largo de los últimos años^(6,8)^, abordarlas con base en las etapas para la transición del cuidado entre terapia intensiva y unidad de hospitalización constituye una perspectiva innovadora. El enfoque permite identificar puntos críticos del proceso y determinar el momento más adecuado para intervenciones específicas, contribuyendo a perfeccionar la calidad de las transiciones y a mitigar los impactos negativos de esa experiencia en el período inmediato posterior al alta.

A partir de lo expuesto, se realizó una búsqueda preliminar en *Joanna Briggs Institute* (JBI) *Evidence Synthesis*, en PubMed^®^ y en *Cumulative Index to Nursing and Allied Health Literature* (CINAHL) y no se identificaron protocolos o revisiones actuales o en curso sobre la temática. Por lo tanto, el objetivo de esta revisión de alcance fue mapear las evidencias disponibles sobre las experiencias de pacientes adultos y familiares en relación con la transición del cuidado entre terapia intensiva y unidad de hospitalización.

## Método

### Tipo de estudio

Se trata de una revisión de alcance, orientada por el JBI^(18)^. Esta revisión tuvo como objetivo responder a la pregunta orientadora: “¿Cuáles son las evidencias en la literatura sobre las experiencias de los pacientes adultos y sus familiares en relación con la transición del cuidado entre UCI y unidad de hospitalización?”. La elaboración de la pregunta se realizó mediante la utilización del acrónimo PCC, siendo P (población) – pacientes adultos y/o familiares – miembro de la familia, cuidador principal o responsable de la toma de decisiones; C (concepto) – experiencia del paciente y/o de los familiares; y C (contexto) – transición del cuidado entre UCI y unidad de hospitalización.

El protocolo de esta revisión fue registrado en *Open Science Framework* (https://osf.io/72khs/), y este informe se presenta en consonancia con lo dispuesto en *Preferred Reporting Items for Systematic Reviews and Meta-Analyses* (PRISMA) *Extension for Scoping Reviews Checklist*^(19)^.

### Criterios de elegibilidad

Se incluyeron estudios primarios, revisiones, disertaciones y tesis que abordaran la experiencia de pacientes adultos, o de sus familiares, sometidos a cuidados en un entorno de cuidados intensivos, independientemente de la etiología del ingreso o del período de hospitalización, y que hubieran sido transferidos a una unidad de hospitalización, considerándose la transferencia entre los diferentes niveles asistenciales dentro de una misma institución, publicados en portugués, inglés o español. No hubo restricción respecto al año de publicación. Se excluyeron aquellos que abordaran la transferencia a unidades de cuidados paliativos, obstétricos o psiquiátricos, debido a las singularidades de esos perfiles de pacientes.

### Fuentes de información

Para la identificación de estudios y descriptores potencialmente relevantes, se realizó una búsqueda inicial en las fuentes de información PubMed® y CINAHL. Las palabras contenidas en el título y en el resumen de los trabajos seleccionados, así como los términos de indización utilizados para describirlos, fueron analizadas para desarrollar una estrategia completa.

La estrategia final, incluidos los términos identificados, se adaptó para cada fuente de información enumerada, es decir, los portales PubMed^®^ y Biblioteca Virtual en Salud y las bases de datos CINAHL, *Cochrane Database of Systematic Reviews*, Embase, *Scopus y Web of Science*. También se realizó una búsqueda en la *Biblioteca Digital Brasileira de Teses e Dissertações* y en la *Networked Digital Library of Theses and Dissertations*.

La búsqueda inicial, así como la pesquisa de los registros con la estrategia final en las diferentes fuentes de evidencia, se realizó entre agosto y septiembre de 2023, con una actualización llevada a cabo del 3 al 9 de septiembre de 2024. Por último, se examinaron las listas de referencias de los estudios incluidos en busca de fuentes adicionales no recuperadas por la estrategia de búsqueda.

### Estrategia de búsqueda

La primera estrategia de búsqueda fue desarrollada utilizando los *Medical Subject Headings* (MeSH) *Terms Patient Discharge*, *Patient Participation*, *Patient-Centered Care*, *Continuity of Patient Care*, *Transitional Care* y *Patient Transfer*. La estrategia final, además de los descriptores mencionados, incorporó sus respectivos sinónimos y los *MeSH Terms Transition in care*, *Critical Care* e *Intensive Care Units*, así como los términos libres *Perception* y *Experience*, combinados mediante los operadores booleanos OR y AND.

Para ilustrar la estrategia final, se presenta en la Figura 1 la búsqueda para el portal PubMed®. La estrategia de búsqueda ajustada para cada fuente de información puede encontrarse en el repositorio de datos SciELO Data (https://doi.org/10.48331/scielodata.PDUSZX). Se destaca que la concepción y el refinamiento de este delineamiento contaron con la colaboración de una bibliotecaria.

**Figura 1-:** Estrategia de búsqueda para el portal PubMed^®^. Porto Alegre, RS, Brasil, 2024

**Fuente de información**	**Estrategia de búsqueda**
PubMed ^®^	(((((((((((((((((((“ *Patient Discharge* ”[MeSH *Terms* ]) *OR* (“ *Discharge, Patient* ”[MeSH *Terms* ])) *OR* (“ *Discharges* , *Patient* ”[MeSH *Terms* ])) *OR* (“ *Patient Discharges* ”[MeSH *Terms* ])) *OR* (“ *Discharge Planning* ”[MeSH *Terms* ])) *OR* (“ *Discharge Plannings* ”[MeSH *Terms* ])) *OR* (“ *Planning* , *Discharge* ”[MeSH *Terms* ])) *OR* (“ *Plannings* , *Discharge* ”[MeSH *Terms* ])) *OR* (“ *Patient Discharge* ”[ *Title* / *Abstract* ])) *OR* (“ *Patient Discharges* ”[ *Title* / *Abstract* ])) *OR* (“ *Discharge Planning* ”[ *Title* / *Abstract* ])) OR (“ *Discharge Plannings* ”[ *Title* / *Abstract* ])) *OR* (((((((((((((((((((((“ *Patient Participation* ”[MeSH *Terms* ]) *OR* (“ *Participation* , *Patient* ”[MeSH *Terms* ])) *OR* (“ *Patient* *Involvement* ”[MeSH *Terms* ])) *OR* (“ *Involvement* ,
PubMed ^®^	*Patient* ”[MeSH *Terms* ])) *OR* (“ *Patient Empowerment* ”[MeSH *Terms* ])) *OR* (“ *Empowerment* , *Patient* ”[MeSH *Terms* ])) *OR* (“ *Patient Participation Rates* ”[MeSH *Terms* ])) *OR* (“ *Participation Rate* , *Patient* ”[MeSH *Terms* ])) *OR* (“ *Participation Rates* , *Patient* ”[MeSH *Terms* ])) *OR* (“ *Patient Participation Rate* ”[MeSH *Terms* ])) *OR* (“ *Patient Activation* ”[MeSH *Terms* ])) *OR* (“ *Activation* , *Patient* ”[MeSH *Terms* ])) *OR* (“ *Patient Engagement* ”[MeSH *Terms* ])) *OR* (“ *Engagement* , *Patient* ”[MeSH *Terms* ])) *OR* (“ *Patient Participation* ”[ *Title* / *Abstract* ])) *OR* (“ *Patient Involvement* ”[ *Title* / *Abstract* ])) *OR* (“ *Patient Empowerment* ”[ *Title* / *Abstract* ])) *OR* (“ *Patient Participation Rates* ”[ *Title* / *Abstract* ])) *OR* (“ *Patient Participation Rate* ”[ *Title* / *Abstract* ])) *OR* (“ *Patient Activation* ”[ *Title* / *Abstract* ])) *OR* (“ *Patient Engagement* ”[ *Title* / *Abstract* ]))) *OR* (((((((((((((((((((((((“ *Patient* - *Centered* *Care* ”[MeSH *Terms* ]) *OR* (“ *Care* , *Patient* - *Centered* ”[MeSH *Terms* ])) *OR* (“ *Patient Centered Care* ”[MeSH *Terms* ])) *OR* (“ *Person* - *Centered* *Care* ”[MeSH *Terms* ])) *OR* (“ *Care* , *Person* - *Centered* ”[MeSH *Terms* ])) *OR* (“ *Cares* , *Person* - *Centered* ”[MeSH *Terms* ])) *OR* (“ *Person* *Centered* *Care* ”[MeSH *Terms* ])) *OR* (“ *Patient* - *Focused Care* ”[MeSH *Terms* ])) *OR* (“ *Care* , *Patient* - *Focused* ”[MeSH *Terms* ])) *OR* (“ *Patient Focused Care* ”[MeSH *Terms* ])) *OR* (“ *Nursing* , *Patient* - *Centered* ”[MeSH *Terms* ])) *OR* (“ *Nursing* , *Patient Centered* ”[MeSH *Terms* ])) *OR* (“ *Patient* - *Centered* *Nursing* ”[MeSH *Terms* ])) *OR* (“ *Patient* *Centered* *Nursing* ”[MeSH *Terms* ])) *OR* (“ *Patient* - *Centered* *Care* ”[ *Title* / *Abstract* ])) *OR* (“ *Patient Centered Care* ”[ *Title* / *Abstract* ])) *OR* (“ *Person* - *Centered* *Care* ”[ *Title* / *Abstract* ])) *OR* (“ *Person* *Centered* *Care* ”[ *Title* / *Abstract* ])) *OR* (“ *Person* - *Centered* *Cares* ”[ *Title* / *Abstract* ])) *OR* (“ *Patient* - *Focused* *Care* ”[ *Title* / *Abstract* ])) *OR* (“ *Patient Focused Care* ”[ *Title* / *Abstract* ])) *OR* (“ *Patient* - *Centered* *Nursing* ”[ *Title* / *Abstract* ])) *OR* (“ *Patient Centered Nursing* ”[ *Title* / *Abstract* ]))) *OR* (((((((((((((“ *Continuity of Patient Care* ”[MeSH *Terms* ]) *OR* (“ *Care Continuity* , *Patient* ”[MeSH *Terms* ])) *OR* (“ *Patient Care Continuity* ”[MeSH *Terms* ])) *OR* (“ *Continuum of Care* ”[MeSH *Terms* ])) *OR* (“ *Care Continuum* ”[MeSH *Terms* ])) *OR* (“ *Continuity of Care* ”[MeSH *Terms* ])) *OR* (“ *Care Continuity* ”[MeSH *Terms* ])) *OR* (“ *Continuity of Patient Care* ”[ *Title* / *Abstract* ])) *OR* (“ *Patient Care Continuity* ”[ *Title* / *Abstract* ])) *OR* (“ *Continuum of Care* ”[ *Title* / *Abstract* ])) *OR* (“ *Care Continuum* ”[ *Title* / *Abstract* ])) *OR* (“ *Continuity of Care* ”[ *Title* / *Abstract* ])) *OR* (“ *Care Continuity* ”[ *Title* / *Abstract* ]))) *OR* ((((((((((“ *Transitional Care* ”[MeSH *Terms* ]) *OR* (“ *Care* , *Transitional* ”[MeSH *Terms* ])) *OR* (“ *Cares* , *Transitional* ”[MeSH *Terms* ])) *OR* (“ *Transitional Cares* ”[MeSH *Terms* ])) *OR* (“ *Transition Care* ”[MeSH *Terms* ])) *OR* (“ *Transition Cares* ”[MeSH *Terms* ])) *OR* (“ *Transitional Care* ”[ *Title* / *Abstract* ])) *OR* (“ *Transitional Cares* ”[ *Title* / *Abstract* ])) *OR* (“ *Transition Care* ”[ *Title* / *Abstract* ])) *OR* (“ *Transition Cares* ”[ *Title* / *Abstract* ]))) *OR* (((((((((((((((((((((((((((((((((((((“ *Patient Transfer* ”[MeSH *Terms* ]) *OR* (“ *Patient Transfers* ”[MeSH *Terms* ])) *OR* (“ *Transfer* , *Patient* ”[MeSH *Terms* ])) *OR* (“ *Transfers* , *Patient* ”[MeSH *Terms* ])) *OR* (“ *Patient Transition* ”[MeSH *Terms* ])) *OR* (“ *Patient Transitions* ”[MeSH *Terms* ])) *OR* (“ *Transition* , *Patient* ”[MeSH *Terms* ])) *OR* (“ *Transitions* , *Patient* ”[MeSH *Terms* ])) *OR* (“ *Care Transition* ”[MeSH *Terms* ])) *OR* (“ *Care Transitions* ”[MeSH *Terms* ])) *OR* (“ *Transition* , *Care* ”[MeSH *Terms* ])) *OR* (“ *Transitions* , *Care* ”[MeSH *Terms* ])) *OR* (“ *Transition of Care* ”[MeSH *Terms* ])) *OR* (“ *Health Care Transition* ”[MeSH *Terms* ])) *OR* (“ *Care Transition* , *Health* ”[MeSH *Terms* ])) *OR* (“ *Care Transitions* , *Health* ”[MeSH *Terms* ])) *OR* (“ *Health Care Transitions* ”[MeSH *Terms* ])) *OR* (“ *Transition* , *Health Care* ”[MeSH *Terms* ])) *OR* (“ *Transitions* , *Health Care* ”[MeSH *Terms* ])) *OR* (“ *Patient Turfing* ”[MeSH *Terms* ])) *OR* (“ *Patient Turfings* ”[MeSH *Terms* ])) *OR* (“ *Turfing* , *Patient* ”[MeSH *Terms* ])) *OR* (“ *Turfings* , *Patient* ”[MeSH *Terms* ])) *OR* (“ *Patient Dumping* ”[MeSH *Terms* ])) *OR* (“ *Dumping* , *Patient* ”[MeSH *Terms* ])) *OR* (“ *Patient Transfer* ”[ *Title* / *Abstract* ])) *OR* (“ *Patient Transfers* ”[ *Title* / *Abstract* ])) *OR* (“ *Patient Transition* ”[ *Title* / *Abstract* ])) *OR* (“ *Patient Transitions* ”[ *Title* / *Abstract* ])) *OR* (“ *Care Transition* ”[ *Title* / *Abstract* ])) *OR* (“ *Care Transitions* ”[ *Title* / *Abstract* ])) *OR* (“ *Transition of Care* ”[ *Title* / *Abstract* ])) *OR* (“ *Health Care Transition* ”[ *Title* / *Abstract* ])) *OR* (“ *Health Care Transitions* ”[ *Title* / *Abstract* ])) *OR* (“ *Patient Turfing* ”[ *Title* / *Abstract* ])) *OR* (“ *Patient Turfings* ”[ *Title* / *Abstract* ])) *OR* (“ *Patient Dumping* ”[ *Title* / *Abstract* ]))) *OR* (“ *Transition in care* ”)) *AND* (((((((“ *Critical Care* ”[MeSH *Terms* ]) *OR* (“ *Care* , *Critical* ”[MeSH *Terms* ])) *OR* (“ *Intensive Care* ”[MeSH *Terms* ])) *OR* (“ *Care* , *Intensive* ”[MeSH *Terms* ])) *OR* (“ *Critical Care* ”[ *Title* / *Abstract* ])) *OR* (“ *Intensive Care* ”[ *Title* / *Abstract* ]) *OR* (((((((“ *Intensive Care Units* ”[MeSH *Terms* ]) *OR* (“ *Intensive Care Unit* ”[MeSH *Terms* ])) *OR* (“ *Unit* , *Intensive Care* ”[MeSH *Terms* ])) *OR* (“ *ICU Intensive Care Units* ”[MeSH *Terms* ])) *OR* (“ *Intensive Care Units* ”[ *Title* / *Abstract* ])) *OR* (“ *Intensive Care Unit* ”[ *Title* / *Abstract* ])) *OR* (“ *ICU Intensive Care Units* ”[ *Title* / *Abstract* ])))) *AND* (( *Perception* [ *Title* / *Abstract* ]) *OR* *(Experience* [ *Title* / *Abstract* ]))

### Selección de las fuentes de evidencia

Los hallazgos se cargaron en el gestor de referencias EndNote *Web* para la eliminación automática de duplicados. A continuación, se utilizó el *software* Rayyan para dar continuidad al proceso, aún con la exclusión manual de los trabajos duplicados.

Los títulos y los resúmenes fueron evaluados por dos revisoras de forma ciega e independiente para la selección en relación con los criterios de elegibilidad especificados. Cualesquiera desacuerdos entre las revisoras durante la selección fueron resueltos por consenso. Cuando no fue posible un acuerdo, se consultó a una tercera investigadora para la decisión final.

### Extracción de los datos

El texto completo de los registros seleccionados fue evaluado detalladamente por dos investigadoras independientes. Se elaboró por las autoras una ficha de extracción de datos, que contenía los siguientes ítems: autor(es), título, periódico, año, volumen, número, página(s), *digital object identifier* o enlace de acceso, país donde se realizó el trabajo, tipo de publicación, objetivo(s), criterio(s) de inclusión y de exclusión, muestra, recolección y análisis de los datos, resultados principales, etapas para la transición del cuidado entre terapia intensiva y unidad de hospitalización y evidencia(s) identificada(s) a partir de la lista de referencias.

Dos investigadoras se familiarizaron con la ficha en una prueba piloto, es decir, al menos dos fuentes fueron consultadas para garantizar la adecuación del formulario al propósito de esta investigación.

Los datos fueron extraídos por una revisora y una segunda investigadora verificó la información recabada. Cualquier desacuerdo entre las investigadoras fue evaluado por una tercera revisora.

### Tratamiento y análisis de los datos

La síntesis de los datos extraídos se organizó en forma de figuras y análisis descriptivo, buscando la alineación con el objetivo y con la pregunta de investigación. Además, se desarrolló un análisis de contenido cualitativo básico, empleando enfoque deductivo^(20)^, para categorizar las experiencias de los pacientes y sus familiares conforme a las etapas para la transición del cuidado entre terapia intensiva y unidad de hospitalización, a saber: evaluación, planificación, ejecución y acompañamiento^(8)^.

### Aspectos éticos

Al tratarse de una revisión basada en datos secundarios, de dominio público y disponibles en la literatura, la evaluación ética es prescindible. No obstante, se destaca que se respetaron los derechos de autor con las debidas citas y referencias de los estudios consultados.

## Resultados

La búsqueda en las bases de datos identificó 7.646 registros y otros siete fueron considerados potencialmente elegibles a partir de las listas de referencias de las fuentes seleccionadas, permaneciendo 30 estudios en la muestra final, conforme se ilustra en la Figura 2.

**Figura 2-:** Diagrama de Flujo PRISMA^(21)^. Porto Alegre, RS, Brasil, 2024

*PubMed^®^ (n = 1.328); Biblioteca Virtual en Salud (n = 878); *Cumulative Index to Nursing and Allied Health Literature* (n = 1.374); *Cochrane Database of Systematic Reviews* (n = 138); Embase (n = 1.555); Scopus (n = 1.817); *Web of Science* (n = 505); *Biblioteca Digital Brasileira de Teses e Dissertações* (n = 13); *Networked Digital Library of Theses and Dissertations* (n = 38)

Las características de los registros incluidos, según año, país, tipo de estudio, objetivo(s) y muestra, se encuentran resumidas en la Figura 3.

**Figura 3-:** Caracterización de los estudios incluidos. Porto Alegre, RS, Brasil, 2024

**Autor(es), año**	**País**	**Tipo de estudio**	**Objetivo(s)**	**Muestra**
Leith, 1999 ^( 22 )^	Canadá	Cualitativo	Describir las percepciones del paciente y sus familiares sobre la transferencia desde una UCI*.	Pacientes (n = 53) y familiares (n = 35)
Odell, 2000 ^( 23 )^	Inglaterra	Cualitativo	Analizar cómo se sienten los pacientes al ser transferidos de la UCI* a la UH ^†^ tras la recuperación de una enfermedad o lesión crítica.	Pacientes (n = 6)
McKinney, et al., 2002 ^( 24 )^	Irlanda del Norte	Cualitativo	Explorar la experiencia vivida por los pacientes en la transferencia de la UCI* a la UH ^†^ .	Pacientes (n = 6)
Chaboyer, et al., 2005 ^( 25 )^	Australia	Cualitativo	Explorar las percepciones individuales y colectivas de un grupo de pacientes y sus familiares sobre la transferencia desde la UCI*.	Pacientes (n = 7) y familiares (n = 6)
Strahan, et al., 2005 ^( 26 )^	Irlanda del Norte	Cualitativo	Explorar y describir las experiencias vividas por los pacientes después de su transferencia desde la UCI*.	Pacientes (n = 10)
Field, et al., 2008 ^( 27 )^	Inglaterra	Cualitativo	Explorar y examinar los relatos de expacientes de UCI* para identificar causas adicionales de estrés por reubicación.	Pacientes (n = 34)
Bench, et al., 2010 ^( 28 )^	Inglaterra	Metasíntesis	Identificar los factores más significativos que impactan la experiencia del paciente en el progreso y la recuperación de una enfermedad crítica durante el primer mes tras el alta de la UCI*, y discutir esos factores en relación con el desarrollo de estrategias eficaces de apoyo al alta de la UCI*.	Registros (n = 10) que contemplan pacientes y familiares
Bench, et al., 2011 ^( 29 )^	Inglaterra	Cualitativo	Recabar la opinión de pacientes, familiares y profesionales de la salud sobre los métodos más eficaces de proporcionar información sobre el alta de la UCI*, el contenido de la información necesaria, los beneficios y limitaciones de la información existente y las posibles implicaciones en términos de recursos.	Pacientes (n = 11), familiares (n = 8) y profesionales de la salud (n = 23)
Forsberg. et al., 2011 ^( 30 )^	Suecia	Cualitativo	Describir las experiencias de las personas que recibieron cuidados en la UCI* y fueron transferidas a una UH ^†^ .	Pacientes (n = 10)
Calatayud, et al., 2013 ^( 31 )^	España	Revisión bibliográfica	Identificar, explorar y presentar críticamente las evidencias existentes sobre cómo los pacientes, familiares y profesionales de enfermería experimentan el proceso de transición de la UCI* a la UH ^†^ , y analizar las posibles intervenciones existentes para el desarrollo de un proceso de transición óptimo.	Registros (n = 23) que contemplan pacientes y familiares
Cullinane, et al., 2013 ^( 32 )^	Inglaterra	Revisión de literatura	Analizar las evidencias sobre la transferencia de pacientes desde la UCI* a la UH ^†^ .	Registros (n = 8) que contemplan pacientes y familiares
Cypress, 2013 ^( 33 )^	Estados Unidos	Revisión de literatura	Revisar sistemáticamente los efectos de la transferencia o del alta de la UCI* hacia unidades de hospitalización médico-quirúrgicas en pacientes adultos gravemente enfermos, sus familiares y enfermeros.	Registros (n = 27) que contemplan pacientes, familiares y profesionales de la salud
Häggström, et al., 2014 ^( 34 )^	Suecia	Método mixto	Investigar la percepción de los familiares sobre la calidad del cuidado durante el proceso de transferencia de un paciente desde una UCI* a una UH ^†^ .	Familiares (n = 65)
Ramsay, et al., 2014 ^( 35 )^	Escocia	Método mixto	Explorar las necesidades psicosociales de los pacientes que reciben el alta de la UCI*, en la medida en que estas son captadas utilizando la teoría de las transiciones y el potencial desarrollo del papel de los servicios de proximidad, acompañamiento y enlace de cuidados intensivos.	Pacientes (n = 20)
Gill, et al., 2016 ^( 36 )^	Canadá	Cualitativo	Explorar las experiencias y perspectivas de los pacientes y de sus familias sobre los cuidados en la UCI* sin la influencia indebida de los proveedores de cuidados o de los investigadores “tradicionales”.	Pacientes y familiares (n = 32)
Stelfox, et al., 2017 ^( 37 )^	Canadá	Cualitativo	Comprender los procedimientos actuales de transferencia y las experiencias de los pacientes y de los profesionales (médicos y enfermeros de la UCI* y de la UH ^†^ ), en una visión de 360 grados, sobre la transferencia de la UCI* a la UH ^†^ , con el fin de informar recomendaciones para mejorar los cuidados	Pacientes (n = 451)
Antonio, et al., 2018 ^( 38 )^	Brasil	Cualitativo	Comprender, desde la perspectiva de los acompañantes, la transición de los pacientes que recibieron el alta de las UCI*.	Familiares (n = 30)
de Grood, et al., 2018 ^( 39 )^	Canadá	Cualitativo	Evaluar las perspectivas de los pacientes y de los proveedores de cuidados sobre barreras y facilitadores para transferencias de alta calidad y recomendaciones para mejorar el proceso de transferencia.	Pacientes, familiares y profesionales de la salud (n = 35)
Koilor, et al., 2018 ^( 40 )^	Estados Unidos	Cualitativo	Caracterizar la experiencia familiar inicial de recuperación de enfermedad crítica traumática.	Familiares (n = 13)
King, et al., 2019 ^( 41 )^	Canadá	Revisión de alcance	Describir qué tipos de apoyo necesitan los pacientes después del alta de la UCI* y de qué forma las necesidades de apoyo difieren a lo largo del *continuum* de recuperación (desde el alta de la UCI* hasta la recuperación a largo plazo basada en la comunidad).	Registros (n = 32) que contemplan pacientes
Herling, et al., 2020 ^( 42 )^	Dinamarca	Cualitativo	Explorar las experiencias de pacientes de la UCI* y sus familiares durante la transición a la UH ^†^ , con el fin de encontrar formas de apoyarlos durante el proceso de transición.	Pacientes (n = 10) y familiares (n = 4)
Op ’t Hoog, et al., 2020 ^( 43 )^	Holanda	Cualitativo	Obtener una comprensión más profunda de las experiencias y necesidades de los familiares de pacientes de UCI* durante la transición de la UCI* a una UH ^†^ .	Familiares (n = 13)
Ghorbanzadeh, et al., 2021 ^( 44 )^	Irán	Cualitativo	Investigar la comprensión y los desafíos que enfrentan los pacientes durante su proceso de transición desde la UCI*.	Pacientes y familiares (n = 8) y profesionales de la salud (n = 14)
Lee, et al., 2021 ^( 45 )^	Corea del Sur	Cualitativo	Obtener una comprensión profunda de la experiencia de transferencia de pacientes de UCI* en Corea del Sur mediante un análisis fenomenológico.	Pacientes (n = 15)
Cuzco, et al., 2022 ^( 46 )^	España	Cualitativo	Describir la experiencia de los pacientes durante la transición de la UCI* a la UH ^†^ .	Pacientes (n = 48)
Zhan, et al., 2022 ^( 47 )^	China	Cualitativo	Explorar las experiencias y necesidades de los cuidadores familiares en relación con los cuidados de transición durante la transferencia desde una UCI* a una UH ^†^ .	Familiares (n = 15)
Cuzco, et al., 2023 ^( 48 )^	España	Cualitativo	Explorar las características de la transición de la UCI* a la UH ^†^ a partir de la experiencia vivida por el paciente, e identificar la terapéutica de enfermería durante la transferencia de los pacientes de la UCI* a la UH ^†^ .	Pacientes (n = 48)
Gullberg, et al., 2023 ^( 49 )^	Suecia	Cualitativo	Describir las experiencias de los pacientes en la preparación para la transferencia desde una UCI* a una UH ^†^ .	Pacientes (n = 14)
Pastor, et al., 2023 ^( 50 )^	España	Cualitativo	Conocer los sentimientos y emociones que emergen de la experiencia de alta de una UCI*, e identificar los factores que desencadenan bienestar y malestar emocional en los pacientes.	Pacientes (n = 20)
Meiring- Noordstra, et al., 2024 ^( 51 )^	Holanda	Cualitativo	Explorar las experiencias de familiares de pacientes internados en UCI* con cuadro agudo en la transición de la UCI* a una UH ^†^ y luego al hogar.	Familiares (n = 12)

*UCI = Unidad de Cuidados Intensivos; ^†^UH = Unidad de Hospitalización

Se identificaron publicaciones de 1999 a 2024, destacándose los años 2023 (n = 4), 2018 (n = 3) y 2013 (n = 3). En cuanto a la procedencia, se evidenciaron estudios conducidos por investigadores del Reino Unido (n = 7), de Canadá (n = 5) y de España (n = 4). En cuanto al tipo de estudio, los autores desarrollaron mayoritariamente investigaciones primarias cualitativas (n = 21) para abordar la temática, contemplando como población prevalente de interés a los pacientes (n = 13), seguida de la díada (pacientes y familiares) (n = 7) y solo familiares (n = 6). Los estudios incluidos fueron conducidos predominantemente por autores con formación en enfermería y medicina, abarcando profesionales asistenciales, gestores, docentes e investigadores.

Los procedimientos de recolección de datos de los estudios incluidos se realizaron en momentos distintos: en la unidad de hospitalización tras el alta de la UCI, pudiendo variar de uno a siete días posteriores a la transferencia^(23-24,26,37,40,42-43,45-48)^; y en el domicilio, por contacto telefónico, videollamada o en el retorno a la institución según combinación, de meses^(25,28,30,35,40,42,49,51)^ a años^(27,29,36)^ después del alta hospitalaria.

Las experiencias de los pacientes y sus familiares, retratadas en las publicaciones mapeadas, categorizadas conforme las etapas para la transición del cuidado entre terapia intensiva y unidad de hospitalización, están representadas en la Figura 4. Una visión general completa de las experiencias está disponible en el repositorio de datos SciELO Data (https://doi.org/10.48331/scielodata.PDUSZX).

**Figura 4:** – Experiencias de pacientes y familiares en la transición del cuidado entre terapia intensiva y unidad de hospitalización. Porto Alegre, RS, Brasil, 2025

*UH = Unidad de Hospitalización; ^†^UCI = Unidad de Cuidados Intensivos

## Discusión

Las experiencias de los pacientes y sus familiares fueron categorizadas, de forma innovadora, a la luz de las etapas descritas para la transición del cuidado entre terapia intensiva y unidad de hospitalización: evaluación para el alta, planificación del alta, ejecución del alta y acompañamiento tras el alta^(8)^. Esta estructura analítica permitió identificar puntos críticos y recurrentes del proceso, destacando la transición como una fase sensible de la hospitalización, marcada por elevada carga emocional y por brechas informacionales transversales a todas las etapas. La ausencia de preparación adecuada para el alta, asociada a deficiencias en la comunicación entre las unidades, se revela como factor que dificulta la continuidad del cuidado y la adaptación de los individuos al nuevo entorno.

Los hallazgos de esta revisión contribuyen a la sistematización de evidencias que otorgan voz a los propios individuos que vivencian la transición, revelando un escenario persistente de fragilidades aún poco superadas: a lo largo de más de dos décadas, predominaron experiencias negativas de pacientes y familiares en ese contexto. Además, los hallazgos señalan direcciones promisorias para investigaciones futuras, orientadas al profundizamiento de la comprensión de las demandas específicas de los individuos, así como a la evaluación del impacto de sus experiencias en la recuperación y en la continuidad del cuidado.

Pacientes y familiares experimentan una transición situacional y organizacional. Situacional, pues vivencian una transición salud-enfermedad desde la admisión, diagnóstico crítico, recuperación y alta de la terapia intensiva; y organizacional, al transitar entre dos unidades con configuraciones distintas, inherentes a sus respectivos niveles asistenciales. Esta transición, por sí sola, expone al individuo a una situación de vulnerabilidad^(1,52)^.

En la etapa de evaluación para el alta, se observa un movimiento creciente entre los profesionales hacia el establecimiento de criterios objetivos para esa decisión, fundamentados en la estabilidad orgánica del individuo y en la capacidad de la unidad receptora. Sin embargo, la visión del paciente y de la familia es poco debatida^(12,53-54)^, corroborando las manifestaciones de falta de involucramiento y distanciamiento respecto de la decisión tomada por el profesional. A diferencia del contexto de alta hospitalaria, que ya cuenta con un instrumento validado y traducido a diversos idiomas para evaluar la preparación en la percepción del paciente^(55-56)^.

Los pacientes críticos generalmente pierden autonomía y se encuentran en una situación de dependencia para las actividades básicas de la vida diaria. El conocimiento y la preparación para el cambio facilitan el empoderamiento del individuo para adaptarse al nuevo contexto y retomar su independencia. La educación en salud se encuentra entre las principales intervenciones de enfermería en ese proceso de transición^(57-58)^. Trabajos que involucran el desarrollo de un modelo^(59)^ o de un paquete de medidas^(60)^ para la transición del cuidado en el alta de la UCI consideran la información como facilitadora de ese proceso, en la medida en que capacita, proporciona herramientas para una participación más activa y promueve relaciones colaborativas.

El intercambio de informaciones graduales, repetidas y apoyadas por material escrito y espacios de escucha para el esclarecimiento de dudas, conducidos de forma respetuosa, son pilares de transiciones exitosas^(8,61-62)^. Se relatan iniciativas para involucrar y capacitar a los individuos para el alta de la UCI^(63-64)^, para la educación centrada en la familia^(65)^ y para los cuidados después de ese momento crítico^(66)^. Sin embargo, en consonancia con los resultados encontrados en esta revisión, otras investigaciones señalan que pacientes y familiares son frecuentemente excluidos de la planificación del alta, recibiendo informaciones insuficientes y divergentes, con términos técnicos y poco tiempo dedicado a esa actividad^(6,8)^.

El intervalo de tiempo ideal para esa planificación aún es desconocido, pero se han considerado por profesionales y gestores de cuidados intensivos^(12)^ períodos inferiores a seis horas y superiores a 48 horas. No obstante, la presión por la disponibilidad de camas^(67-68)^, aliada a la elevada carga de trabajo^(69)^, con frecuencia crea un entorno agitado de tal forma que las oportunidades instructivas, muchas veces, son menospreciadas^(6,69)^.

Las experiencias de los pacientes y sus familiares fueron categorizadas, de forma innovadora, a la luz de las etapas descritas para la transición del cuidado entre terapia intensiva y unidad de hospitalización: evaluación para el alta, planificación del alta, ejecución del alta y acompañamiento tras el alta^(8)^. Esta estructura analítica permitió identificar puntos críticos y recurrentes del proceso, destacando la transición como una fase sensible de la hospitalización, marcada por elevada carga emocional y por brechas informacionales transversales a todas las etapas. La ausencia de preparación adecuada para el alta, asociada a deficiencias en la comunicación entre las unidades, se revela como factor que dificulta la continuidad del cuidado y la adaptación de los individuos al nuevo entorno.

Los hallazgos de esta revisión contribuyen a la sistematización de evidencias que otorgan voz a los propios individuos que vivencian la transición, revelando un escenario persistente de fragilidades aún poco superadas: a lo largo de más de dos décadas, predominaron experiencias negativas de pacientes y familiares en ese contexto. Además, los hallazgos señalan direcciones promisorias para investigaciones futuras, orientadas al profundizamiento de la comprensión de las demandas específicas de los individuos, así como a la evaluación del impacto de sus experiencias en la recuperación y en la continuidad del cuidado.

Pacientes y familiares experimentan una transición situacional y organizacional. Situacional, pues vivencian una transición salud-enfermedad desde la admisión, diagnóstico crítico, recuperación y alta de la terapia intensiva; y organizacional, al transitar entre dos unidades con configuraciones distintas, inherentes a sus respectivos niveles asistenciales. Esta transición, por sí sola, expone al individuo a una situación de vulnerabilidad^(1,52)^.

En la etapa de evaluación para el alta, se observa un movimiento creciente entre los profesionales hacia el establecimiento de criterios objetivos para esa decisión, fundamentados en la estabilidad orgánica del individuo y en la capacidad de la unidad receptora. Sin embargo, la visión del paciente y de la familia es poco debatida^(12,53-54)^, corroborando las manifestaciones de falta de involucramiento y distanciamiento respecto de la decisión tomada por el profesional. A diferencia del contexto de alta hospitalaria, que ya cuenta con un instrumento validado y traducido a diversos idiomas para evaluar la preparación en la percepción del paciente^(55-56)^.

Los pacientes críticos generalmente pierden autonomía y se encuentran en una situación de dependencia para las actividades básicas de la vida diaria. El conocimiento y la preparación para el cambio facilitan el empoderamiento del individuo para adaptarse al nuevo contexto y retomar su independencia. La educación en salud se encuentra entre las principales intervenciones de enfermería en ese proceso de transición^(57-58)^. Trabajos que involucran el desarrollo de un modelo^(59)^ o de un paquete de medidas^(60)^ para la transición del cuidado en el alta de la UCI consideran la información como facilitadora de ese proceso, en la medida en que capacita, proporciona herramientas para una participación más activa y promueve relaciones colaborativas.

El intercambio de informaciones graduales, repetidas y apoyadas por material escrito y espacios de escucha para el esclarecimiento de dudas, conducidos de forma respetuosa, son pilares de transiciones exitosas^(8,61-62)^. Se relatan iniciativas para involucrar y capacitar a los individuos para el alta de la UCI^(63-64)^, para la educación centrada en la familia^(65)^ y para los cuidados después de ese momento crítico^(66)^. Sin embargo, en consonancia con los resultados encontrados en esta revisión, otras investigaciones señalan que pacientes y familiares son frecuentemente excluidos de la planificación del alta, recibiendo informaciones insuficientes y divergentes, con términos técnicos y poco tiempo dedicado a esa actividad^(6,8)^.

El intervalo de tiempo ideal para esa planificación aún es desconocido, pero se han considerado por profesionales y gestores de cuidados intensivos^(12)^ períodos inferiores a seis horas y superiores a 48 horas. No obstante, la presión por la disponibilidad de camas^(67-68)^, aliada a la elevada carga de trabajo^(69)^, con frecuencia crea un entorno agitado de tal forma que las oportunidades instructivas, muchas veces, son menospreciadas^(6,69)^.

Las barreras de comunicación y la preparación inadecuada son factores relacionados con el diagnóstico de enfermería “síndrome de estrés por reubicación”, definido como una alteración fisiológica y/o psicosocial asociada a la transferencia de un entorno a otro, caracterizada, entre otros, por preocupación, inseguridad, ansiedad y miedo^(70)^, sentimientos consistentemente mencionados en la muestra seleccionada. Investigadores validaron una herramienta de autoinforme para evaluar el estrés por reubicación en pacientes tras la transferencia de la UCI, con el propósito de identificar problemas psicológicos durante el cambio y auxiliar a los profesionales en la elaboración de cuidados centrados en la persona, con vistas a minimizar las perturbaciones asociadas al período de transición^(71)^.

La transferencia de cuidados a pie de cama para la *ejecución del alta*^(69,72)^ fue identificada como experiencia positiva por pacientes y familiares y viene siendo referida como estrategia favorable para la transición del cuidado. En contrapartida, los retrasos debidos a la indisponibilidad de camas en la unidad de hospitalización^(73-75)^, transferencias en fines de semana^(75-77)^, en los cambios de turno^(77)^ o nocturnas^(77-78)^ contribuyeron a la insatisfacción de los individuos y se han asociado a desenlaces negativos.

En cuanto a la comunicación entre los equipos de la UCI y de la unidad de hospitalización, tanto los profesionales^(79)^ como los pacientes y familiares señalaron fragilidades en ese proceso. Los estudios muestran que, a pesar de las orientaciones, la comunicación carece de estandarización y, comúnmente, los datos transmitidos quedan a criterio de los profesionales de la UCI^(8,80-82)^. La literatura también resalta que las prácticas actuales están por debajo de lo ideal, con discrepancias entre la información transmitida por el equipo intensivista y las necesidades de quienes la reciben en la unidad de hospitalización^(8,69,77,80,82)^.

Los profesionales de la UCI frecuentemente sobreestiman la capacidad del equipo receptor^(13-14,79,82)^, que, a su vez, percibe esa transición como una gran responsabilidad. Esta percepción se intensifica por la recepción de pacientes inestables, con historiales complejos, tratamientos poco claros, transferidos precozmente y con preparación insuficiente para abandonar de forma segura el entorno de la terapia intensiva. El desafío se torna aún mayor cuando el equipo receptor se siente inexperto o carece de cualificación para atender ese perfil de paciente^(67,69)^, fragilidad percibida tanto por los pacientes como por los familiares. Enfermeros de unidades de hospitalización sugirieron como estrategias de mejora el contacto previo con enfermeros intensivistas para el intercambio de información y la posibilidad de realizar un período en la UCI para observar los procedimientos efectuados y profundizar la comprensión sobre el cuidado crítico^(67)^. Esta práctica también beneficia a pacientes y familiares al posibilitar un primer contacto con los profesionales del equipo receptor, antes de la transferencia, favoreciendo la continuidad y la humanización del cuidado^(83)^.

Entre las publicaciones analizadas, la reducción abrupta y mal comunicada del cuidado asistencial destacó como una experiencia estresante. Cuando no hay orientación sobre las diferencias esperadas en la intensidad del soporte entre las unidades, la percepción del individuo puede distorsionarse, llevando a comparaciones irreales. Además, existe un desajuste entre la dependencia de los cuidados intensivos recibidos en la UCI y la necesidad de autosuficiencia para ejecutar las mismas tareas en la unidad de hospitalización, sin tiempo suficiente para esa adaptación. Los familiares frecuentemente relatan, como adversidades relacionadas con el alta de la terapia intensiva, la pérdida de información, la reducción en el monitoreo y la sensación de transferencia precoz^(11)^. Para mejorar la calidad de la transición y garantizar la seguridad del paciente, es fundamental implementar prácticas como la reducción gradual del nivel de cuidado todavía en la UCI, incluyendo el retiro de dispositivos invasivos innecesarios, la retirada progresiva de la monitorización multiparamétrica y la evaluación de la movilidad^(6,84-85)^.

Asimismo, la presencia y el involucramiento de la familia son imprescindibles en el proceso de alta de la UCI. Muchos individuos vivencian el alta de la terapia intensiva con nivel y/o contenido de conciencia alterados, con déficit cognitivo y/o de comunicación importante o dependientes para actividades básicas de la vida diaria, y los familiares actúan como un vínculo de soporte emocional e informacional, ayudando a su adecuación a la nueva fase de recuperación y, en múltiples ocasiones, asumiendo papeles decisorios en relación con conductas y tratamientos^(6,8,86)^. Buscan activamente ser reconocidos como parte esencial en el cuidado de su familiar^(87)^.

Programas de *seguimiento tras el alta* de la UCI han sido implementados de diversas formas. Equipos de transición del cuidado monitorizan al paciente en la unidad de hospitalización por al menos 24 horas después del alta de la terapia intensiva^(88-90)^, mientras que servicios de enfermería de enlace realizan actividades orientadas a la seguridad, manejo de vías aéreas, prevención de infecciones y caídas, cuidados de la piel y educación y soporte a pacientes y familiares^(91-92)^. Aunque revisiones sistemáticas y metaanálisis indican evidencias limitadas sobre la efectividad de estas intervenciones en la reducción de readmisiones y mortalidad^(93-94)^, los familiares informaron que sus necesidades fueron atendidas y consideran valiosa la actuación de los enfermeros de enlace, especialmente por el soporte ofrecido durante la transición^(95)^.

Un estudio destacó los beneficios de un Grupo de Cuidados Posintensivos, en el cual enfermeros de la UCI ofrecieron cuidados adicionales a los pacientes más vulnerables en la unidad de hospitalización. Estos cuidados se dirigieron a las necesidades físicas y psicológicas de los pacientes, incluyendo visitas más frecuentes, cuando fuese necesario, lo que promovió mayor confianza y reducción de la ansiedad tanto en los pacientes como en sus familiares. Las principales actividades del grupo se concentraron en la prevención, con la detección precoz de señales de deterioro, la mitigación de eventos adversos y la promoción del intercambio de conocimientos entre los equipos, con foco en el mejor interés del paciente^(96)^.

Adicionalmente, los 30 estudios analizados fueron publicados de manera equilibrada a lo largo del tiempo y conducidos por distintos perfiles profesionales, actuantes en la asistencia, gestión, docencia e investigación. Esta distribución evidencia una demanda constante de producción de conocimiento sobre el tema, así como el interés compartido entre distintos segmentos de la práctica y de la ciencia por comprender y cualificar este proceso. Aunque la predominancia recae en autores de enfermería y medicina, la complejidad del fenómeno investigado exige abordajes integrados y el desarrollo de estudios futuros que incorporen la perspectiva del equipo multidisciplinario.

Más de la mitad de los estudios fueron desarrollados en el continente europeo, lo que refleja una concentración de la producción científica en pocos países y resalta la necesidad de explorar esta temática en otras realidades. Revisiones de alcance que abordaron la transición del cuidado entre UCI y unidad de hospitalización, de pacientes adultos^(6)^ y de neonatales, pediátricos y adultos^(8)^, también señalaron a Europa y América del Norte como los principales orígenes de la mayoría de las publicaciones incluidas.

Otro tópico relevante sobre las características de los estudios es la variabilidad en los períodos de recolección de datos adoptados por los investigadores. No se describe el intervalo de tiempo ideal; al parecer, los autores consideran períodos clave para captar impresiones de interés contextuales. Por ejemplo, para comprender cómo los pacientes y sus familias vivencian el entorno de la terapia intensiva y su impacto en la recuperación, los datos fueron recolectados en la primera semana tras el alta de la UCI^(97)^; ya para investigar los efectos de la realidad virtual en la salud mental, en la calidad percibida y en la satisfacción después del tratamiento intensivo entre sobrevivientes de COVID-19, la información fue recolectada hasta seis meses después del alta hospitalaria^(98)^.

Entre las limitaciones de esta revisión de alcance se encuentran la restricción de idioma y la inaccesibilidad al texto completo de cuatro registros, lo que puede haber resultado en una muestra incompleta y menos representativa. Igualmente, aunque la extracción de datos fue realizada de forma sistemática por dos revisoras, es posible que no haya sido totalmente precisa y exhaustiva.

Esta revisión contribuye al avance del conocimiento científico para el área de salud y de enfermería al reunir evidencias provenientes de diferentes contextos y temporalidades y ampliar la visibilidad de las etapas de la transición del cuidado entre terapia intensiva y unidad de hospitalización desde la perspectiva de pacientes y familiares. La representación creada (Figura 4) puede subsidiar actividades de educación permanente en salud, al sintetizar y operacionalizar el conocimiento disponible, con énfasis en las fragilidades identificadas en cada etapa del proceso. Los hallazgos implican la necesidad de perfeccionar las estrategias actualmente adoptadas, mediante intervenciones estructuradas y oportunas, con miras a cualificar la continuidad asistencial y reducir las experiencias negativas aún recurrentes a lo largo de esa trayectoria.

## Conclusión

La transición del cuidado entre terapia intensiva y unidad de hospitalización es un proceso complejo y multifacético, frecuentemente asociado a desafíos que afectan a pacientes, familiares y equipos asistenciales. Las experiencias descritas en las publicaciones mapeadas destacan ese momento como un evento significativo de la hospitalización, a menudo acompañado de impacto emocional. La falta de información fue identificada en todas las etapas del proceso, con relatos recurrentes de falta de preparación y percepciones de abandono, especialmente en casos de transferencia repentina. Además, la comunicación inadecuada entre las partes involucradas resultó en desajustes asistenciales, dificultando la adaptación de los individuos y comprometiendo la transición.

En este contexto, la educación en salud se destaca como una de las principales intervenciones de enfermería para reducir las brechas identificadas. Ofrecer informaciones claras y oportunas, explicar las diferencias esperadas entre los niveles asistenciales y adoptar una comunicación estructurada son estrategias primordiales. Se reafirma, como contribución relevante de esta revisión, la categorización de las experiencias de pacientes y familiares entre las etapas de la transición del cuidado entre terapia intensiva y unidad de hospitalización, la cual proporciona una visualización clara y accesible de los principales puntos críticos del proceso. Esta sistematización podrá emplearse como recurso estratégico en actividades de educación permanente en salud, al fomentar la traducción del conocimiento en la práctica e indicar aspectos que demandan atención en cada etapa.

Asimismo, es esencial promover una cultura organizacional con responsabilidades claramente definidas entre las categorías profesionales involucradas en la preparación del paciente y de la familia. Este enfoque debe sustituir prácticas aisladas y minimizar la dependencia de acciones subjetivas, fortaleciendo un entorno de cuidado colaborativo.

Avanzar en la comprensión de este tema exige el desarrollo de estrategias basadas en evidencias, incluyendo modelos integrados de transición con comunicación estandarizada, programas educativos para preparar a pacientes y familiares antes de la transferencia e instrumentos validados para medir percepciones sobre preparación, satisfacción y seguridad durante el proceso. Las investigaciones futuras pueden contribuir a identificar prácticas que promuevan autonomía, empoderamiento y seguridad y a comprender mejor el impacto de la transición del cuidado en las experiencias vividas. De esta forma, será posible no solo reducir el estrés y la vulnerabilidad asociados a ese momento, sino también garantizar una atención equitativa, eficiente y centrada en la persona.

## Declaración de Disponibilidad de Datos

El conjunto de datos de este artículo está disponible en la página de la RLAE en el repositorio SciELO Data, en el enlace https://doi.org/10.48331/scielodata.PDUSZX
